# Fungal carbonatogenesis process mediates zinc and chromium removal via statistically optimized carbonic anhydrase enzyme

**DOI:** 10.1186/s12934-024-02499-7

**Published:** 2024-08-27

**Authors:** Naira A. Awadeen, Marwa Eltarahony, Sahar Zaki, Amany Yousef, Samy El-Assar, Hadeel El-Shall

**Affiliations:** 1 Microbiology Department, Faculty of Dentistry, Pharos University, Alexandria, Egypt; 2 Evironmental Biotechnology Department, Genetic Engineering and Biotechnology Research Institute (GEBRI), City of Scientific Research and Technological Applications (SRTA-City), New Borg El-Arab City, Alexandria, 21934 Egypt; 3 Department of Botany and Microbiology, Faculty of Science, Alexandria University, Alexandria, Egypt

**Keywords:** Microbial induced calcium carbonate precipitation, Bioremediation, Heavy metals, Response surface methodology, And wastewater treatment

## Abstract

**Introduction:**

With rapid elevation in population, urbanization and industrialization, the environment is exposed to uncontrolled discharge of effluents filled with broad-spectrum toxicity, persistence and long-distance transmission anthropogenic compounds, among them heavy metals. That put our ecosystem on the verge or at a stake of drastic ecological deterioration, which eventually adversely influence on public health. Therefore, this study employed marine fungal strain *Rhodotorula sp.* MZ312369 for Zn^2+^ and Cr^6+^ remediation using the promising calcium carbonate (CaCO_3_) bioprecipitation technique, for the first time.

**Results:**

Initially, Plackett–Burman design followed by central composite design were applied to optimize carbonic anhydrase enzyme (CA), which succeeded in enhancing its activity to 154 U/mL with 1.8-fold increase comparing to the basal conditions. The potentiality of our biofactory in remediating Zn^2+^ (50 ppm) and Cr^6+^ (400 ppm) was monitored through dynamic study of several parameters including microbial count, CA activity, CaCO_3_ weight, pH fluctuation, changing the soluble concentrations of Ca^2+^ along with Zn^2+^ and Cr^6+^. The results revealed that 9.23 × 10^7^ ± 2.1 × 10^6^ CFU/mL and 10.88 × 10^7^ ± 2.5 × 10^6^ CFU/mL of cells exhibited their maximum CA activity by 124.84 ± 1.24 and 140 ± 2.5 U/mL at 132 h for Zn^2+^ and Cr^6+^, respectively. Simultaneously, with pH increase to 9.5 ± 0.2, a complete removal for both metals was observed at 168 h; Ca^2+^ removal percentages recorded 78.99% and 85.06% for Zn^2+^ and Cr^6+^ remediating experiments, respectively. Further, the identity, elemental composition, functional structure and morphology of bioremediated precipitates were also examined via mineralogical analysis. EDX pattern showed the typical signals of C, O and Ca accompanying with Zn^2+^ and Cr^6+^ peaks. SEM micrographs depicted spindle, spherical and cubic shape bioliths with size range of 1.3 ± 0.5–23.7 ± 3.1 µm. Meanwhile, XRD difractigrams unveiled the prevalence of vaterite phase in remediated samples. Besides, FTIR profiles emphasized the presence of vaterite spectral peaks along with metals wavenumbers.

**Conclusion:**

CA enzyme mediated Zn^2+^ and Cr^6+^ immobilization and encapsulation inside potent vaterite trap through microbial biomineralization process, which deemed as surrogate ecofriendly solution to mitigate heavy metals toxicity and restrict their mobility in soil and wastewater.

## Background

Heavy metals are widely used in modern industrial production. However, due to the incomplete waste treatment after production process, a large amount of sewage and solid wastes containing such heavy metals are discharged into the environment, causing serious environmental pollution [1]. Metal ions persist in the environs due to the bioaccumulation affinity of living organisms and their restricted ability to metabolize them into less toxic forms. Once they absorbed into the cell of living organisms, they bind to vital components, for instance nucleic acids, structural proteins and enzymes, impairing thereby numerous essential metabolic functions [2].

Several industrial processes such as textile dyeing, tanneries, metal electroplating, battery manufacturing units, mining, metallurgy, pigment/paint, galvanizing steel, paper bleaching and corrosion inhibition wastewater treatment plants are responsible for increasing Chromium (Cr) and Zinc (Zn) concentrations as particles released into the terrestrial atmosphere or as wastewater discharged into the ground or waterways. Bearing in mind, Cr (VI) is a well-known carcinogen, teratogen, mutagen, and have a toxic effect on all living systems with strong migration ability, whose toxicity and mutagenicity are exceeded that of Cr (III) by 100 times [3]. Regrettably in similar way, exposure to certain levels of Zn, even within a short time, can cause severe health issues such as stomach cramps, anemia, nausea, vomiting, reducing of HDL cholesterol and pancreas damage [2, 4]. Hence, the World Health Organization (WHO) recommended a maximum acceptable Zn concentration in drinking water of 5.0 mg/L, and the allowable limit of Cr (VI) in natural water is 0.05 mg/L [5]. Based on the above mentioned facts, the treatment of water and soil contaminated with toxic metals is a challenging issue to find technically, economic, feasible, ecofriendly and effective procedures. Several technical approaches have been proposed to control metals toxicity, among them, carbon adsorption, chemical precipitation, electrochemical treatment, ion exchange, reverse osmosis, and membrane separation. However, these conventional methods are expensive, energy-intensive, and produce toxic sludge that requires special and additional handling [6]. Remarkably, the biological remediation pathways are deemed being environmentally friendly to eliminate toxic metals, with unique advantages of low cost and high efficiency, especially at low concentrations [7]. Interestingly, a plethora of former studies have proved the feasibility of bioremediation using microorganism to detoxify metals and transform them to less toxic forms [7–9]. Microbial remediation mechanisms mainly include bioaccumulation, biosorption, bioleaching, biotransformation, and biomineralization, which basically rely on metal-microbe interactions [10–12]. Notably, the microbial mineralization is described as one of the most promising bioremediation techniques that could be executed through many microbial metabolic processes, which generates numerous biominerals. About 60 and more type of biominerals have been produced via microbial mineralization process either directly or indirectly such as phosphate, oxalate, and carbonate that could encompass numerous metal ions, like Mg^+^, Mn^++^, Fe^+++^, Ca^++^, etc. [13, 14].

Microbial induced calcium carbonate precipitation (MICP) is a unique biomineralization process by which different microbes induce bio-mineralization of CaCO_3_ in the presence of dissolved calcium (Ca^2+^) and carbonate (CO_3_^2−^) ions [15]. This process has been investigated as a potential method for the removal of heavy metals from contaminated water sources. It mediates the mineralization of heavy metals from the ionic soluble state into a stable precipitated solid form, thus, reduces the mobility and toxicity of those hazardous metals [16, 17]. The bio-precipitation of calcium carbonate could be implemented via either an autotrophic or heterotrophic pathways, while the later seems to be more popular. The autotrophic biosynthesis of CaCO_3_ involves different mechanisms such as methanogenesis, aerobic photosynthesis and anaerobic photosynthesis [18]. On the other hand, three main categories of microorganisms enhance bio-calcification technique heterotrophically. The first category induces the reduction of sulphate by sulphate reducing bacteria (SRB) [19]. The second category comprises microorganisms that engage in nitrogen cycle by the subsequent ways: (A) ammonification of amino acids, (B) denitrification and C) urea hydrolysis [20]. The third category enhances the reversible transformation of carbon dioxide (CO_2_) to bicarbonate through carbonic anhydrase enzyme (CA) [17].

CA has been associated in MICP, CO_2_ sequestration and subsequently lessens greenhouse effect. It catalyzes the reversible CO_2_ hydration and dehydration of HCO^3−^ through Ping-Pong mechanism (i.e., 2-stage); utilizing zinc bound hydroxide ion found in the enzymes active site. Through MICP process, HCO^3−^ can interact with Ca^2+^ under alkaline conditions, which were precipitated as CaCO_3_ crystals [15, 17]. Thus, this enzyme possesses the ability to accelerate CO_2_ uptake, besides carbonate rock dissolution at the same time. Notably, it is anticipated that CA reaction will afford the key vital molecules for the formation of CaCO_3_ when proceeding in the forward direction [21]. In addition, CA has many other functions, as it has been established to be imperative to plant growth, stomatal development, responses to various stresses, as well as participating in photosynthetic light reactions, while capturing the atmospheric CO_2_ [22, 23]. On the other hand, CA is requested for rapid processes in several organisms, commonly transport processes. For instance, it is necessary in the removal of CO_2_ from lungs for the synthesis of eye secretions. Additionally, CA keeps optimum level of HCO^3−^ and CO_2_ in the body, which both used as substrate for many enzymatic reactions. Moreover, CA has vital role in blood as it retains acid–base balance, helps in homeostasis the physiological pH, and also participates in respiration and ion transport [24]. However, the majority of studies concerning with calcification technology addressed urea hydrolysis and few researches were interested in denitrification mechanism [25]. Nonetheless, no study, till our knowledge, documented the recruitment of carbonic anhydrase enzyme in heavy metal removal through MICP process.

Remarkably, the employed microorganism in any application must be effective in its performance, however, other secondary properties (e.g., biosafety, proliferation requirements/conditions, formulation options, bioreactor application, recovery, etc.) are just as or even more influential. Therefore, noteworthy shed the light to the characteristic features of unicellular fungi (i.e., yeasts), which appeal scientific community for their recruiting in wide spectrum of applications, with less complications elicitation than filamentous fungi or bacteria. They are relatively abundant in various ecosystems, easily culturing, wide nutritional requirements, facultative anaerobes, short generation time relative to multicellular fungi, higher biomass yield and better adaptability with higher metabolites productivity per biomass unit. Let alone, their higher stability and complexity of genome organization, biofilm forming capacity, superior metal bioaccumulation potentials, enhanced tolerance to adverse circumstances with multiple detoxification mechanisms, which situated them as a platform for innovative applications [26]. Strikingly, all previously mentioned traits of yeasts triggered them a promising biogenic factory for multifaceted applications in all aspects of biotechnology, which were symbolized by rainbow code, namely red biotechnology [27, 28], yellow biotechnology [29, 30], gray/white biotechnology [31–33], gold biotechnology [34, 35], green biotechnology [36–39].

Intriguingly, the capability of yeasts to generate calcite through MICP was recorded previously [11, 12]. Besides, their versatile metabolic diversity that enable them to exhibit a noticeable survival performance in extreme environments such as concrete, limestone, mortar, marble and granite were also documented [9]. Despite several remediation mechanisms exerted by different yeast stains for purifying the environment from pollutants, the remediation through MICP proved its superior effectiveness. Wherein, the remediated metals entrapped inside durable CaCO_3_ matrix; displaying thereby more potent trap than surface adsorption, internal accumulation or even hydroxide precipitates, which exposed to re-dissolution in water and generation of soluble anionic hydroxyl complexes [40]. Additionally, Eltarahony et al. [40] found that there were no dissociation or dissolution of remediated metals sequestered in the calcareous trap under acidic pH (pH 3.8 ± 0.045), reflecting the stabilization of immobilized metals with low chance to release in ambient ecosystems even under highly acidic conditions such as acidic rain.

Nevertheless, no study to the best of our knowledge invested the capacity of yeast in remediating heavy metals through MICP process, in particular via enhanced performance of CA enzyme. While Barbero et al. [41] had successfully edited the genes encoding carbonic anhydrase and mineralization peptides in yeast, which would significantly enhance its ability to induce CaCO_3_ precipitation. Based on the previous background, the present study, for the first time, aimed to eliminate Zn^2+^ and Cr^6+^ using the carbonate precipitation efficiency of carbonic anhydrase-producing yeast isolate. The core of this study focused on the statistical optimization to maximize the performance of carbonic anhydrase, which thereafter, enhanced MICP process and elevated Zn^2+^ and Cr^6+^ elimination efficacy^.^ Finally, the removal of examined metals was confirmed through various mineralogical analyses (i.e., energy dispersive X-ray spectrometry (EDX), scanning electron microscopy (SEM), X-ray diffraction (XRD), and Fourier transform infrared spectroscopy (FTIR) of Zn^2+^ and Cr^6+^ enclosed in CaCO_3_ deposits.

## Materials and methods

### Microorganism, and cultural conditions

A water sample was collected from Marsa Allam, Red sea governerate, Egypt. Directly after sampling, isolation and screening of biomineralizing microbes were performed. Initially, the samples were inoculated in Basal media (B4) containing glucose (21) g/L, ca-acetate (18 g/L), NaNO_2_ (1.5) at pH 7.0 ± 0.2, 30 ℃ [40]. Out of 15 microbial isolate, one isolate was selected based on its higher carbonatogenesis capability. The strain was stored in glycerol (20%, v/v) at − 20 ℃ for the forthcoming investigations.

### Morphological and cultural characterization

The morphology and dimensions of the selected isolate were determined from photomicrographs utilizing scanning electron microscopy (SEM) (JEOL JEM-1230, Japan- Faculty of Science- Alexandria University). For colony characterization, the cells were cultivated under both aerobic and anaerobic conditions on YPD agar (Yeast Extract, 10.0 g, Peptone 20.0 g, Dextrose, 20.0 g, Agar, 15.0 g) and incubated at 30 ℃. [42]. About 0.5 McFarland equivalents to about 1.5 × 10^8^ CFU/mL was inoculated in 250 mL Erlenmeyer flasks containing 100 mL of the YPD medium and incubated at 30 °C in an orbital shaker (STUART SI500) at 150 rpm. After the incubation period, the cells were centrifuged at 10.000 X*g* for 20 min at 4 ℃ and the harvested cells were used for further tests.

### Biochemical and physiological characteristics

Tests of catalase, oxidase, and amylase were performed as reported by Nunes et al. [43]. For physiological experiments, the optimum pH, growth temperature and aeration conditions were determined. The freshly prepared culture was inoculated on YPD at various initial pH: 5–9. The buffers (0.1 mM) used in pH experiments were citrate–phosphate (pH: 2–6), phosphate (pH 7.0), and Tris-hydrochloride (pH 8.5) [44]. For temperature, the cultivated YPD medium was incubated at 4 ℃, 10 ℃, 20 ℃, 30 ℃, 40 ℃, 50 ℃ and 60 ℃, at pH 7. For aeration conditions, the inoculated YPD medium (pH 7) were incubated at 30 ℃ in static incubator and shaker incubator at different agitation speed 50, 100, 150 and 200 rpm.

### Molecular identification

The selected isolate was identified using 18S rRNA gene sequencing. The genomic DNA of the selected isolate was extracted from overnight pure cultures and 18S rRNA gene was amplified using18S primers [45], then the purified PCR product was sequenced as described elsewhere [46]. The phylogenetic affiliation was inquired by applying BLASTn analysis to determine the similarities with their available GenBank database sequences. Its generated sequence was submitted to the GenBank to obtain its corresponding accession number. For multiple alignment and phylogenetic tree construction, the software package MEGA- 6 was employed.

### Assay of carbonic anhydrase enzyme (CA)

The CA was determined in the fungal pellets that were collected by centrifugation at 10,000 X*g* for 20 min. Then the pellets were dissolved in phosphate buffer (pH 7.0), mixed well and disrupted by ice cold TSE buffer (10 mM Tris HCl, 100 mM NaCl, and 1.0 mM EDTA at a pH of 7.8). The reaction was incubated for 30 min at 30 ℃ with good vortex every 10 min. The cell debris and unbroken cell were removed by centrifugation at 10,000 X*g* for 3 min, while the supernatant represents the crude enzyme [47]. CA activity was assessed by calculating the micromole of *p*-nitrophenol liberated from *p*-NPA (*p*-nitrophenyl acetate). The reaction mixture composed of (in order of addition):825 μL of Phosphate buffer (50 mM, pH 7.5), 175 μL of the substrate stock solution (*p*-NPA, 10 mM in isopropanol). The blank reactions contain the media without yeast culture. Then, 25 μL of an enzyme was added to initiate the reaction. The reaction mixture was incubated at 37 ℃ in a water bath for 5 min [48]. The amount of *p*-nitrophenol released was estimated at 410 nm using a spectrophotometer [47]. One unit of CA activity correlates to the quantity of enzyme that enhances the formation of 1 μmol of p-nitrophenol per minute under standard assay conditions.

### Experimental design and statistical analysis

In order to optimize the concentration of all media components simultaneously and not by changing a single factor, statistical experimental designs were applied in two steps, namely, (i) Plackett- Burman Design “PBD” to determine the important factors affecting CaCO_3_ precipitation and CA activity, (ii) Central Composite Design (CCD) as a type of Response Surface Methodology (RSM) to infer the exact concentrations of important factors that achieve the maximum calcium carbonate (CaCO_3_) precipitation and CA activity.

### Determination of significant parameters by Plackett-Burman design (PBD)

PBD is the design that intended for screening and identifying the controlled experimental parameters (nutritional, environmental and incubation conditions) based on their main effect on CA enzyme activity and CaCO_3_ weight. PBD investigates (n) variables which are expressed at two levels, high (+) and low (−). Herein, a total of 7 (n) variants were studied at 2-level concentrations in 12 experimental matrices, as shown in Table 1. Each experiment was done in triplicate and CA activity and CaCO_3_ weight were assessed as response [40]. The Plackett–Burman experimental design is built on the first order model (Eq. 1):

1 $${\text{Y }} = \beta {\text{O }} + \sum {\beta {\text{iXi}}}$$

where, Y is the response or dependent variable (CaCO_3_ weight and CA activity); it will always be the variable we aim to predict, βo is intercept of the model, βi is the linear coefficient, and Xi is the level of the independent variable. The statistical analysis output will be used to calculate the significance of the variables depending on their nature; and their positive or negative effects on CA enzyme activity and the weight of CaCO_3_.

**Table 1:** Matrix of Plackett–Burman experimental design, variables and their levels along with experimental and predicted response values for screening of significant processes variables affecting CaCO_3_ weight and CA activity

Run order	Glucose	Ca-acetate	NaNO_2_	Peptone	Inoculum size	Incubation time	ZnCl_2_	Experimental CaCO_3_ weight	Predicted CaCO_3_ weight	St. Resid.	Experimental CA activity	predicted ca activity	St. Resid.
1	1	1	− 1	1	1	− 1	1	1.13	1.105	0.55	116	113.48	0.54
2	− 1	− 1	− 1	1	1	1	− 1	0.42	0.365	1.2	43	37.38	1.2
3	1	1	− 1	1	− 1	− 1	− 1	0.9	0.925	− 0.55	92.5	95.02	− 0.54
4	− 1	1	1	− 1	1	− 1	− 1	0.3	0.245	1.2	30.8	25.15	1.2
5	− 1	− 1	1	1	1	− 1	1	0.02	0.0667	− 1.02	2	6.75	− 1.01
6	− 1	1	1	1	− 1	1	1	0.19	0.2317	− 0.91	19.5	23.78	− 0.91
7	1	− 1	− 1	− 1	1	1	1	0.67	0.7033	− 0.73	68.8	72.18	− 0.72
8	1	1	1	− 1	1	1	− 1	0.55	0.605	− 1.2	56.5	62.15	− 1.2
9	1	− 1	1	1	− 1	1	− 1	0.28	0.2467	0.73	28.7	25.28	0.73
10	1	− 1	1	− 1	− 1	− 1	1	0.28	0.225	1.2	28.7	23.08	1.2
11	− 1	− 1	− 1	− 1	− 1	− 1	− 1	0.1	0.1633	− 1.38	10.2	16.72	− 1.39
12	− 1	1	− 1	− 1	− 1	1	1	0.55	0.5083	0.91	56.5	52.22	0.91

Variable	Unit	Coded levels/experimental values		
		− 1	0	1
Glucose	g/L	3	10	15
Ca-acetate	g/L	3	10	15
NaNO_2_	g/L	1	5	7
Peptone	g/L	3	10	15
Inoculum Size (0.5 McFarland)	%	1	5	10
Incubation period	Days	3	5	7
ZnCl_2_	mg/L	0.01	0.1	0.3

### Central composite design (CCD)

To determine the optimal levels of the most interesting variables that were detected by PBD and to infer their interactions, RSM was applied using CCD. Five levels (− 2, − 1, 0, + 1, + 2) of four different variables (i.e., glucose, sodium nitrite, calcium acetate and inoculum size) were studied in 31 experiments as listed in Table 2. Along with each experiment both CA activity and CaCO_3_ weight were determined. Each Experiment was performed in triplicate and the mean was calculated for subsequent statistical analysis [40]. Considering statistical estimation, the relationship between the coded and actual values is represented by Eq. 2:

2 $$\text{Xi }=\text{ Ui }-\text{ Ui}0/\Delta \text{Ui}$$

where Xi is the coded value of the ith variable, Ui is the real value of the ith variable; Ui0 is the real value of the ith variable at the centre point and ΔUi is the step change of variable. The second order polynomial structured described in Eq. 3:

3 $$\text{Y }=\upbeta 0 +\upbeta 1\text{X}1 +\upbeta 2\text{X}2 +\upbeta 3\text{X}3 +\upbeta 11\text{X}11 +\upbeta 22\text{X}22 +\upbeta 33\text{X}33 +\upbeta 12\text{X}1\text{X}2 +\upbeta 13\text{X}1\text{X}3 +\upbeta 23\text{X}2\text{X}3$$

where: Y is the predicted response; X1, X2, X3 are input variables that affect the response variable Y; β0, intercept; β1, β2 and β3 linear coefficients; β11, β22 and β33, squared or quadratic coefficients β12, β13, and β23 interaction coefficients.

**Table 2:** Central composite design matrix of CaCO_3_ weight and CA activity along with the predicted responses and variables concentrations

Run order	Glucose	Calcium acetate	NaNO_2_	Inoculum size	Experimental CaCO_3_ weight	Predicted CaCO_3_ weight	Experimental CA activity	Predicted CA activity
					(g/50 mL)		(U/mL)	
1	− 1	1	− 1	− 1	0.15	0.152	15.4	16.636
2	− 1	− 1	− 1	1	0.19	0.27	19.5	28.419
3	0	0	0	− 2	0.09	0.137	9.5	13.061
4	0	0	0	0	0.97	0.913	99.5	88.95
5	1	1	1	1	1.2	1.153	123	117.67
6	0	0	0	0	0.98	0.913	100.5	88.95
7	1	− 1	− 1	− 1	0.1	0.225	10.2	24.103
8	− 1	1	− 1	1	0.2	0.333	20.5	34.823
9	0	0	2	0	0.67	0.682	68.7	71.884
10	1	1	− 1	1	0.88	0.934	90.3	96.419
11	− 1	1	1	1	0.83	0.867	85.2	88.399
12	− 1	1	1	− 1	0.61	0.546	62.6	55.887
13	1	1	− 1	− 1	0.59	0.543	60.5	56.757
14	0	0	0	0	0.86	0.913	74.75	88.95
15	− 1	− 1	1	1	0.45	0.445	46.2	45.07
16	0	0	− 2	0	0.55	0.428	63.72	48.308
17	1	− 1	1	− 1	0.13	− 0.055	13.3	-5.897
18	0	0	0	0	0.79	0.913	81	88.95
19	− 1	− 1	− 1	− 1	0.1	0.095	10.2	10.657
20	0	0	0	2	1	0.843	100.6	84.811
21	− 2	0	0	0	0.3	0.212	30.8	21.278
22	1	1	1	− 1	0.54	0.622	55.5	63.683
23	0	− 2	0	0	0.09	0	9.2	-0.506
24	1	− 1	− 1	1	0.6	0.611	61.5	63.34
25	2	0	0	0	0.65	0.628	66.7	63.994
26	0	2	0	0	0.76	0.74	78	75.478
27	0	0	0	0	0.83	0.913	85.2	88.95
28	0	0	0	0	1.09	0.913	92.4	88.95
29	0	0	0	0	0.87	0.913	89.3	88.95
30	1	− 1	1	1	0.31	0.47	31.8	47.666
31	− 1	− 1	1	− 1	0.02	0.129	2	12.982

Variable	Unit	Coded levels/Experimental Values				
		− 2	− 1	0	1	2
Glucose	g/L	5	10	15	20	25
NaNO_2_	g/L	0.25	0.5	1	2	3
Calcium acetate	g/L	5	7.5	10	15	20
Inoculum size	%	3	5	10	20	30

### Statistical analysis and verification of the model

The statistical software Minitab 14.0 (Minitab Inc., Pennsylvania, USA software) was used for establishing the experiment designs “matrices” and subsequent statistical analysis of PBD and CCD data (regression analysis and ANOVA). Further, the relationship between the response and variables was also illustrated graphically by three-dimensional surface plots (3D) and two-dimensional contour plots (2D). In addition, the optimizer tool was used to predict the optimum level of experimental factors. Under predicted optimized conditions, the model was validated through comparing CA enzyme activity and the weight of CaCO_3_ with that obtained from the basal conditions [40]**.**

### Bioremediation of Zn^2+^and Cr^6+^ in MICP process

#### Minimal inhibitory concentration test

Firstly, the metal toxicity experiment was performed to decide the minimum inhibitory concentration (MIC) of Zn^2+^ and Cr^6+^. Different concentrations (800, 400, 200, 100, 50, 25 and, 12.5 ppm) of Zn^2+^ (ZnCl_2_) and Cr^6+^ (K_2_Cr_2_O_7_) were examined in YPD agar media inoculated with 0.5 McFarland scale. The inoculated plates were incubated at 30 ℃ for 48 h in triplicate. Accordingly, 150 mL of optimized mineralizing media in 500 mL flasks supplemented with 50 ppm of Zn^2+^ and 400 ppm of Cr^6+^ (½ MIC) were inoculated by 10^8^ CFU/mL. The flasks were incubated in a rotary shaker (150 rpm) at 30 ℃ for 7 days. At the same time, two controls were run in parallel; abiotic controls or negative controls which was devoid from fungal inoculum and the biotic controls contained inoculated biomineralization media without heavy metals [40].

### Monitoring of chemical changes during Zn^2+^and Cr^6+^ removal

Changes in solution chemistry during MICP were assessed in the form of cell count, carbonic anhydrase (CA) activity, pH and, concentrations of soluble Ca^2+^, Zn^2+^ and Cr^6+^. During the biomineralization mechanism, the culture media was drawn at a constant time interval to evaluate the previous parameters. The cell number (CFU/mL) was detected by the pour plate method; the CA activity and pH were assessed as described previously. The concentrations of soluble Ca^2+^, Zn^2+^ and Cr^6+^ were estimated by an inductively coupled plasma optical emission spectrometer (Agilent ICP-OES 5110DVD) (Central Lab, Alexandria university). All experiments were performed in triplicate and the mean values were considered. At the end of the incubation period, all precipitates were centrifuged at 10,000 X*g* for 20 min, then washed, dried and subjected to mineralogical analysis [40]. The bioremediation efficiency of examined strains was calculated as a percentage representing the differences between the initial and final concentrations of Ca^2+^, Zn^2+^ and Cr^6+^ in the supernatant.

### Mineralogical and morphological analysis

To evaluate the identity, morphology, microstructure and chemical constituents of the precipitated samples (positive control and remediated deposits with Zn^2+^ and Cr^6+^), X-ray diffraction (XRD), Energy dispersive X-ray spectroscopy (EDX), scanning electronic microscopy (SEM) and Fourier transform infrared spectroscopy (FTIR) were utilized. XRD was used to identify the precipitated minerals **(**Bruker MeaSrv D2-208219, Germany-Central Lab, Faculty of science, Alexandria University), which were irradiated with Cu Kα (λ = 0.15406 nm), generated at 30 kV and 30 mA at a scan rate of 2°/min for 2θ values over a wide range of Bragg angles 10° ≤ 2θ ≤ 80. Microchemical analysis of the samples was achieved using EDX analyzer combined with SEM (JEOL JSM 6360LA, Japan). The morphological features of deposits were visualized using SEM (JEOL JSM 6360LA, Japan- Advanced Technologies and New Materials Research Institute (ATNMRI) SRTA-City) at an accelerating voltage of 20 kV. To scrutinize the accompanying functional groups of CaCO_3_ crystals, FTIR was performed by Shimadzu FTIR-8400 S, Japan with a resolution of 4 cm^−1^. Sample preparation prior to FTIR analysis began with mixing the bioprecipitated CaCO_3_ crystals with KBr followed by pulverization to powder and pressing into discs. The spectrum was scanned with a range of 4000 and 400 cm^−1^.

## Results and discussion

### Screening of CaCO_3_ producing microorganisms

**Fig. 1:**
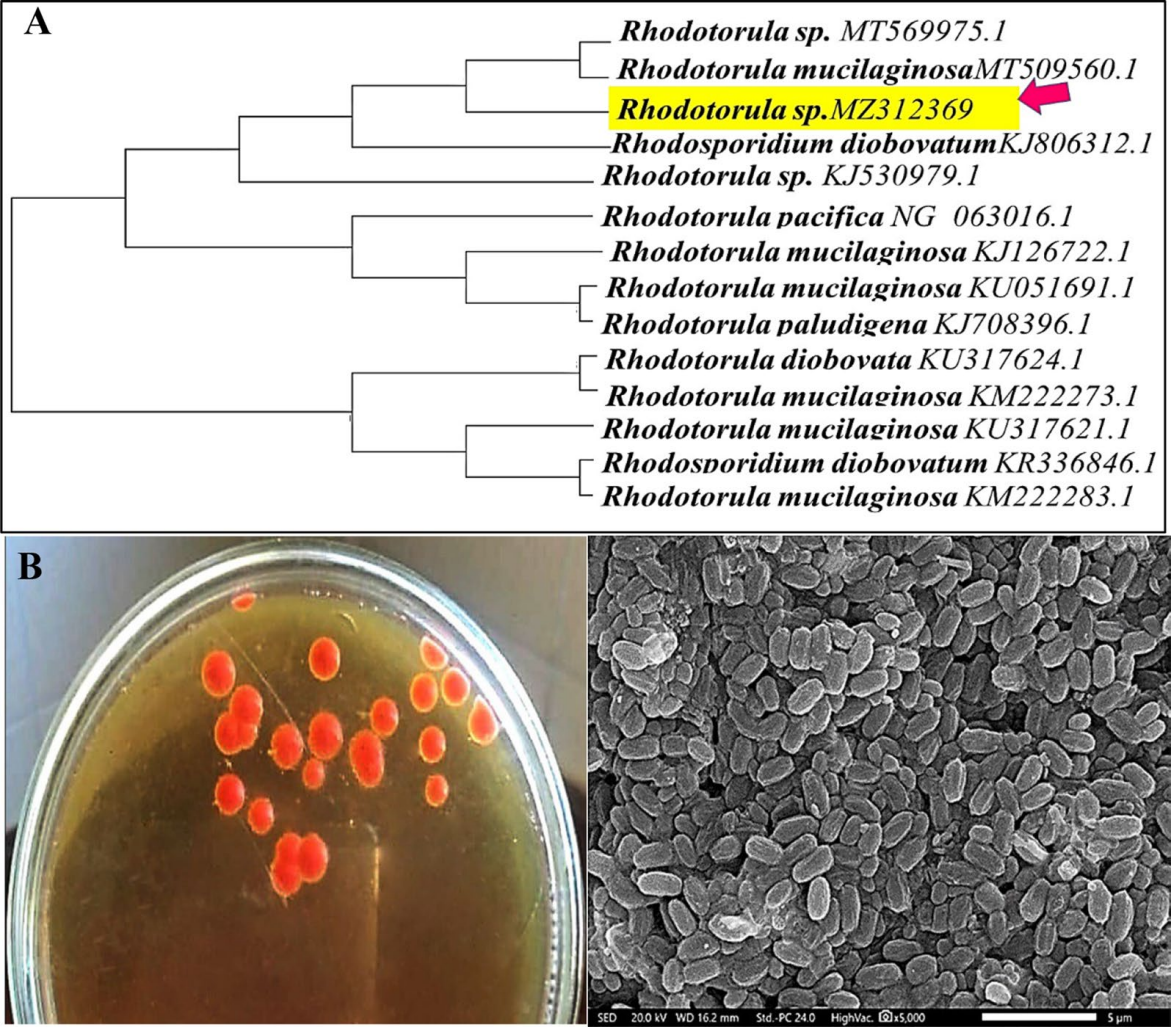
Neighbor-joining dendrogram (**A**), cultural characteristics on YPD agar plate (**B**) and morphological features of *Rhodotorula sp*. MZ312359 cells as examined by SEM analysis

Actually, the signal for the MICP approach was monitored through the presence of solid deposits on B4-agar medium [40]. Herein, the isolate under study was selected among 15 isolates as precipitated the highest amount of CaCO_3_ in B4-broth medium comparing to the others. Subsequently, the molecular characterization was performed by identifying the partial 18S rRNA gene sequence (≈500 bp), which revealed 99% sequence resemblance with all species of the genus *Rhodotorula* and less than that with other genera. Remarkably, the accepted taxonomic affiliation standard is considered at sequence similarity greater than 98% between reference and inquired strains as mentioned by Nunes et al. [43]. Accordingly, it was identified as *Rhodotorula sp*. Its nucleotide sequence was submitted to GenBank with the accession number of MZ312359. The phylogenetic position was constructed as illustrated in Fig. 1A, the members of genus *Rhodotorula* are affiliated to the basidiomycetic fungi and well-known for their ability to biosynthesize a diverse range of valuable biomolecules, including carotenoids, lipids, enzymes, and polysaccharides [49]. However, it was not studied before as calcifying microbe, till our acquaintance.

### Cultural, physiological and morphological characterization

On YPD agar, the colony appeared spherical, pigmented, flat or slightly raised with smooth borders as illustrated in Fig. 1B. It characterized by orange pigments which seemed to be carotenoid that acts as defensive mechanism against oxidative stress [50]. It exhibited aerobic and anaerobic growth (i.e., facultative anaerobic) but at a faster pace in oxygen consuming conditions. As a unicellular eukaryot, it was visulaized as non-motile elongated spherical or oval cells shape under SEM, their length ranged from 1.5 to 2.8 μm and its width lied between 0.5 and 0.9 μm Fig. 1C. Besides, the examined *Rhodotorula sp.* MZ312369 showed various biochemical activities as illustrated in Table 3. It was capable of growing on a broad temperature range varies from 10 ℃ to 40 ℃ with optimum at 25–30 ℃, below and above this range the growth adversely affected as harmonized with Allahkarami et al. [51]. Whereas, its growth in different pH ranged from 6 to 9 reflected its optimal performance at an initial pH of 7.0–8.0. Similarly, the physiological and biochemical characters of our isolate agreed with that examined by Nunes [43].

**Table 3:** Biochemical properties of *Rhodotorula sp*. MZ312359

Biochemical test	Result	Biochemical test	Result
Catalase	+	Nitrate test	+
Oxidase	−	Fluconazole	−
Bile esculin	+	Nystatin	+
Urease	+	Ketoconazole	+
Glucose fermentation	+	Gelatin liquification	−
Sucrose fermentation	+	Acid production	−
Fructose fermentation	+	Starch formation	−
Lactose fermentation	+	Maltose fermentation	−
Galactose fermentation	−	Xylose fermentation	+
Mannitol fermentation	−	Mannose fermentation	−
Glycerol fermentation	−		

### Determination of significant parameters by Plackett–Burman (PBD)

The Plackett–Burman experimental design is a useful tool for the rapid evaluation and screening of the significant nutritional and environmental parameters affecting on examined responses [52–54], which were CA activity and CaCO_3_ weight. The influence of 7 independent factors on the overall carbonatogenic process (i.e., CA activity and the weight of CaCO_3_) was detected in 12 experimental runs. The results exhibited a wide variation in enzyme activity from 2 to 116 U/mL and in the CaCO_3_ precipitates from 0.02 to 1.13 g as shown in Table 1. Such variation reflected the leverage of nutritional and environmental factors combination on CA activity and CaCO_3_ weight. It is important to find out the adequacy and significance of the model, hence, the data of CA activity and CaCO_3_ weight were analyzed to declare multiple linear regression analysis and analysis of variance (ANOVA). Generally, the coefficient with lower probability P-value (≤ 0.05) indicates that the corresponding factor is significant and possesses a high effect on response (CaCO_3_ weight & CA activity), while a coefficient with probability P-value exceeds 0.05 reveals the negligible effect on response. Besides, the sign of the examined variable’s coefficient gives an insinuation about the influence of variable’s concentration on response. Namely, the positive sign of coefficient indicates the higher response upon using the higher value/concentration of the corresponding variable and vice versa. Out of Table 4, the calculated confidence level and p- values manifested that glucose, Ca-acetate, NaNO_2_ and inoculum size were the significant parameters on MICP process.

**Table 4:** Estimated effects, regression coefficients and corresponding *P-*values for the first order equation model of CaCO_3_ weight and CA activity optimized by PBD

Term	Estimated Effects and Coefficients for CaCO_3_ weight						Estimated Effects and Coefficients for CA Enzyme					
	Effect	Coef	SE Coef	T	P	Confidence level %	Effect	Coef	SE Coef	T	P	Confidence level %
Constant		0.4492	0.02293	19.59	0	100		46.1	2.347	19.64	0	100
Glucose	0.3717	0.1858	0.02293	8.11	0.001	99.9	38.2	19.1	2.347	8.14	0.001	99.9
Ca-acetate	0.3083	0.1542	0.02293	6.72	0.003	99.7	31.73	15.87	2.347	6.76	0.002	99.8
NaNO_2_	− 0.3583	− 0.1792	0.02293	-7.81	0.001	99.9	− 36.8	− 18.4	2.347	-7.84	0.001	99.9
Peptone	0.0817	0.0408	0.02293	1.78	0.15	85	8.37	4.18	2.347	1.78	0.149	85.1
Inoculum size	0.1317	0.0658	0.02293	2.87	0.045	95.5	13.5	6.75	2.347	2.88	0.045	95.5
Incubation days	− 0.0117	− 0.0058	0.02293	− 0.25	0.812	18.8	− 1.2	− 0.6	2.347	− 0.26	0.811	18.9
Zncl_2_	0.0483	0.0242	0.02293	1.05	0.351	64.9	4.97	2.48	2.347	1.06	0.35	65
R^2^ = 0.9788 R^2^(adj) = 0.9417%							R^2^ = 0.9789% R^2^(adj) = 0.9421%					

The model coefficient of determination R^2^ and adjusted-R^2^ were 0.9788%, 0.9789% and 0.9417%, 0.9421% for CaCO_3_ weight and CA activity, respectively, which implies that 97.88% and 97.89% of the variation in the data were illustrated by the model Table 4. While there were just 2.12% & 2.11% chance that could occur because of the noise. This again ensured a satisfactory adjustment and a good relation between the observed and the predicted values. As mentioned in Mojtaba and Fardin [55], the closer R^2^ is to 1, the better the estimation of regression equation fits the sample data**.** However, the residuals were studentized and their values were in the range of ± 2, which fall in reasonable range as denoted by Anuar et al. [56]. Additionally, as revealed by ANOVA, the first-order equations representing the optimum of CA activity and CaCO_3_ weight as a function of the studied independent factors were expressed as follows in Eqs. 4 and 5:

4 $$\mathbf{Y}(\mathbf{C}\mathbf{A}\mathbf{a}\mathbf{c}\mathbf{t}\mathbf{i}\mathbf{v}\mathbf{i}\mathbf{t}\mathbf{y}) = 46.1 + 19.1\text{glucose }+ 15.87\text{ Ca}-\text{acetate }- 18.4\text{ NaN}{\text{O}}_{2} + 4.18\text{ protease peptone }+ 6.75\text{ inoculum Size \% }- 0.6\text{ incubation day }+ 2.48\text{ ZnC}{\text{l}}_{2}$$

5 $$\mathbf{Y}\left(\mathbf{C}\mathbf{a}\mathbf{C}{\mathbf{O}}_{3}\mathbf{W}\mathbf{e}\mathbf{i}\mathbf{g}\mathbf{h}\mathbf{t}\right)= 0.4492 + 0.1858\text{ glucose }+ 0.1542\text{ Ca}-\text{acetate}- 0.1792\text{ NaN}{\text{O}}_{2} + 0.0408\text{ protease peptone }+ 0.0658\text{ inoculum Size \%}- 0.0058\text{ incubation day }+ 0.0242\text{ ZnC}{\text{l}}_{2}$$

### Central composite design for optimizing CA activity and CaCO_3_ weight

RSM is a mean of statistics that depends on key statistical concepts, randomization, replication and duplication. Remarkably, it facilitates the optimization via statistically valid studies of mutual interactions between variables across a scope of values. Besides, it predicts the optimum performance conditions in the least trial numbers and concluded the individual / interactive impacts of the measured variables on response [57, 58]. The goal of the RSM trials was to get a more accurate evaluation of the optimal operating conditions for the factors screened from PBD at a five-level (− 2, − 1, 0, + 1, + 2) to achieve the maximum CA activity and CaCO_3_ weight. Herein, thirty-one experimental trials with different combinations of glucose, sodium nitrite, calcium acetate concentrations, and inoculum size were investigated. As observed in Table 2, the different coded and actual levels of the four independent parameters and response in each run were illustrated. The results demonstrated a considerable variation in CA activity and CaCO_3_ weight, which recorded the maximum value with 1.2 g and 123 U/mL for CaCO_3_ weight and CA activity, respectively at trial 5. Whereas, the minimum obtained yields were 0.02 g and 2 U/mL for CaCO_3_ weight and CA activity, respectively, at trial 31.

### Regression and analysis of variance (ANOVA)

Multiple regression analysis was employed to statistically analyse the data of CaCO_3_ weight and CA activity as tabulated in Table 5, which also included the values of R^2^, adjusted- R^2^, the coefficient estimates, probability P-value, lack-of-fit, linear, quadratic and interactions impacts as well. As noticed, the values of R^2^, which determine the effectiveness of the polynomial regression model, assessed by 0.931 and 0.927. Their values proved that 93.1% and 92.7% of variation in CaCO_3_ weight and CA activity, respectively, were impacted by the independent variables and only 6.9% and 7.3% could not be explained in the view of models. Besides, the Adj-R^2^ values were quantified as 0.87 and 0.862, which emphasized the model significance. Notably, the small difference between R^2^ and adjusted- R^2^ reflects the good coordination between the actual experimental values and the predicted values of both responses; thus, the models of the current study were optimal within the range of experimental factors to predict an efficient CaCO_3_ weight and CA activity. In addition, the positive coefficient values pointed out that the linear effect of all variables, quadratic effect and mutual interactions effect of some factors exhibited synergistic leverage in CA activity and CaCO_3_ weight (i.e., their higher values enhance CA activity and CaCO_3_ weight). While the other factors, which displayed negative coefficient values signifies their higher impact on carbonatogenesis process at their negative value. For instance, the positive sign of glucose coefficient indicated a positive influence on the weight of CA activity and CaCO_3_ at high concentrations, whereas with more increasing in its concentration, it inhibited the overall process, as evident from negative coefficient of the squared term.

**Table 5:** Estimated effects, regression coefficients and corresponding P-values of second order polynomial model for CaCO_3_ weight and CA activity optimized by CCD

Term		CaCO_3_ weight				CA activity			
		Coef	SE Coef	T	P	Coef	SE Coef	T	P
Constant		0.912857	0.04778	19.106	0	88.95	4.884	18.214	0
Linear effects	Glucose	0.104167	0.0258	4.037	0.001	10.6792	2.637	4.049	0.001
	Ca acetate	0.185	0.0258	7.17	0	18.9958	2.637	7.202	0
	NaNO_2_	0.063333	0.0258	2.454	0.026	5.8942	2.637	2.235	0.04
	Inoculum size	0.176667	0.0258	6.847	0	17.9375	2.637	6.801	0
Quadratic effects	(Glucose)^2^	− 0.123214	0.02364	− 5.212	0	− 11.578	2.416	− 4.792	0
	(Ca-acetate)^2^	− 0.135714	0.02364	− 5.741	0	− 12.866	2.416	− 5.325	0
	(NaNO_2_)^2^	− 0.089464	0.02364	− 3.785	0.002	− 7.2135	2.416	-2.985	0.009
	(Inc.size)^2^	− 0.105714	0.02364	− 4.472	0	− 10.0035	2.416	4.14	0.001
Interaction effects	glucose*Ca acetate	0.065	0.0316	2.057	0.056	6.6688	3.23	2.064	0.056
	Glucose* NaNO_2_	− 0.07875	0.0316	− 2.492	0.024	− 8.0812	3.23	− 2.502	0.024
	Glucose * Inc. Size	0.0525	0.0316	1.661	0.116	5.3688	3.23	1.662	0.116
	Ca-acetate* NaNO_2_	0.09	0.0316	2.848	0.012	9.2313	3.23	2.858	0.011
	Ca-acetate*Inc. Size	0.00125	0.0316	0.04	0.969	0.1062	3.23	0.033	0.974
	NaNO_2_* Inc. Size	0.035	0.0316	1.108	0.284	3.5813	3.23	1.109	0.284
S = 0.1264	R-Sq = 93.1% R-Sq(adj) = 87%					R-Sq = 92.7% R-Sq(adj) = 86.2%			

For interactive terms, the interaction between (glucose & Ca-acetate), (glucose & inoculum size), (Ca-acetate & inoculum size) and (NaNO_2_ & inoculum size) were described as insignificant as given by P-value, which exceeded 0.05. While the other relations, namely glucose & NaNO_2_ and Ca-acetate & NaNO_2_ were both significant. Meaning that they can act as limiting factors and any little difference in their values will alter vividly the CA activity and CaCO_3_ weight as well [59]. Table 5 informed that the relation influence between (glucose & ca-acetate), (glucose & inoculum size), (Ca-acetate & NaNO_2_), (Ca-acetate & inoculum size) and (NaNO_2_ & inoculum size) were positive (i.e., synergistic effect) as CA activity and CaCO_3_ weight increase with increasing in both factors. While, antagonistic effect appeared to be prevailing between glucose and NaNO_2_. That means the effect of higher level of one parameter increases CaCO_3_ precipitation process with lower level of another examined parameter. Generally, the regression models found in this study was highly significant as denoted by the low P-values with 0.000; the linear and quadratic effects appear to be predominant over interactive effect as observed in Table 6. In addition, as unveiled by ANOVA, Lack-of-fit assessed by 0.259 and 0.149 for CaCO_3_ weight and CA activity, respectively, which indicated their insignificance and reflecting the robustness of both models and their accuracy. Moreover, the analysis of the data developed from RSM generally considers a second order polynomial equation, which explains the relation among the response variable(s) and the factors. In this equation the linear, the interactions and quadratic effects of each factor on the response variable(s) are determined. The second-order polynomial equation which defines the predicted response formulated as follows Eqs. 6 and 7:

**Table 6:** ANOVA for quadratic polynomial model of calcium carbonate weight and CA activity

Source	CaCO_3_ weight						CA activity					
	DF	Seq SS	Adj SS	Adj MS	F	P	DF	Seq SS	Adj SS	Adj MS	F	P
Regression	14	3.4441	3.4441	0.246	15.4	0	14	33,714.9	33,714.9	2408.21	14.42	0
Linear	4	1.9272	1.9272	0.4818	30.15	0	4	19,953.2	19,953.2	4988.29	29.88	0
Square	4	1.1568	1.1568	0.2892	18.1	0	4	9975.3	9975.3	2493.83	14.94	0
Interaction	6	0.3602	0.3602	0.06	3.76	0.016	6	3786.5	3786.5	631.08	3.78	0.015
Residual error	16	0.2557	0.2557	0.016			16	2671.2	2671.2	166.95		
Lack-of-fit	10	0.1899	0.1899	0.019	1.73	0.259	10	2135.6	2135.6	213.56	2.39	0.149
Pure error	6	0.0657	0.0657	0.011			6	535.6	535.6	89.27		
Total	30	3.6998					30	36,386.2				

6 $$\mathbf{Y}\left(\mathbf{C}\mathbf{A}\mathbf{a}\mathbf{c}\mathbf{t}\mathbf{i}\mathbf{v}\mathbf{i}\mathbf{t}\mathbf{y}\right)= 0.912857 + 0.104167\text{ glucose }+0.185\text{ Ca}-\text{acetate }+ 0.063333\text{ NaN}{\text{O}}_{2}+0. 176667\text{ inoculum size }- 0.123214 {\left(\text{glucose}\right)}^{2} - 0.135714 {\left(\text{Ca}-\text{acetate}\right)}^{2} - 0.089464 {\left({\text{NaNO}}_{2}\right)}^{2}- 0.105714 {\left(\text{inoculum size}\right)}^{2} + 0.065\text{ glucose}*\text{Ca}-\text{acetate }- 0.07875\text{ glucose}*{\text{NaNO}}_{2} + 0.0525\text{ glucose}*\text{ inoculum size }+0.09\text{ Ca}-\text{acetate}*{\text{NaNO}}_{2}+ 0.00125\text{ Ca}-\text{acetate}*\text{Inc}.\text{size }+ 0.035\text{ NaN}{\text{O}}_{2}*\text{ inoculum size}.$$

7 $$\mathbf{Y}(\mathbf{C}\mathbf{a}\mathbf{C}{\mathbf{O}}_{3}\mathbf{w}\mathbf{e}\mathbf{i}\mathbf{g}\mathbf{h}\mathbf{t}) = 0.912857 + 0.104167\text{ glucose }+0.185\text{ Ca}-\text{acetate }+ 0.063333\text{ NaN}{\text{O}}_{2} + 0. 176667\text{ inoculum size }- 0.123214{ (\text{glucose})}^{2} - 0.135714 {(\text{Ca}-\text{acetate})}^{2}- 0.089464 {(\text{NaNO}2)}^{2} - 0.105714 {(\text{inoculum size})}^{2} + 0.065\text{ glucose}*\text{Ca}-\text{acetate }- 0.07875\text{ glucose}*\text{ NaN}{\text{O}}_{2} + 0.0525\text{ glucose}*\text{inoculum size }+0.09\text{ Ca}-\text{acetate }*\text{ NaN}{\text{O}}_{2}+ 0.00125\text{ Ca}-\text{acetate}*\text{ inoculum size }+ 0.035\text{ NaN}{\text{O}}_{2}*\text{ inoculum size}.$$

### Graphical demonstrations of the response surface model

**Fig. 2:**
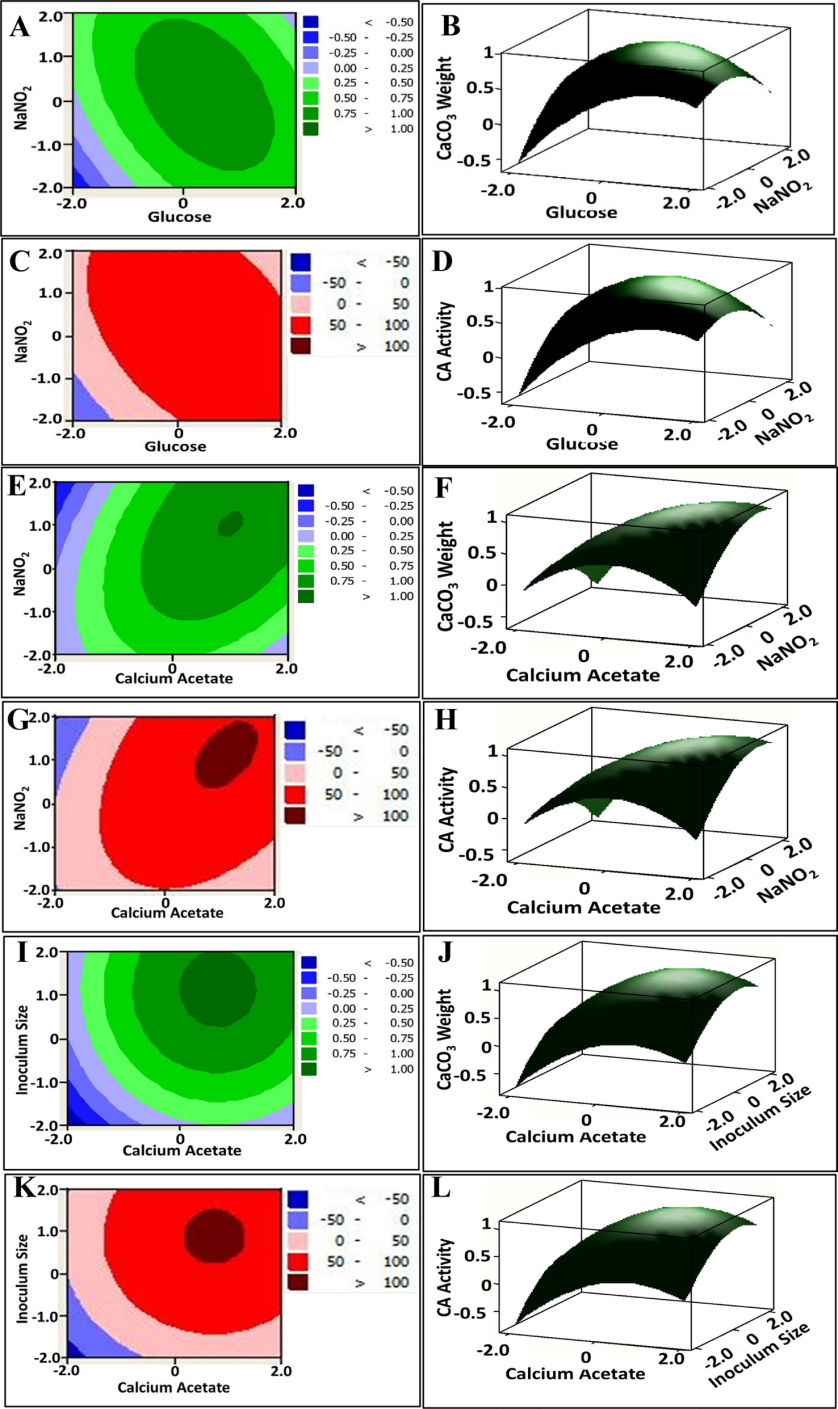
Contour Plots (**A**, **C**, **E**, **G**, **I**, **K**) and Surface plots (**B**, **D**, **F**, **H**, **J**, **L**) for CaCO_3_ weight and CA activity of *Rhodotorula sp*. MZ312359 showing the interactive effects of some variables

The three-dimensional surface (3D) and two-dimensional contour (2D) plots were generated to understand the interaction of the variables and responses (CA activity and CaCO_3_ weight) and also predict the ideal level of each variable for maximal response; such plots are a graphical representation of the model equations achieved in the regression analysis [59]. The response surface plot is a 3-D graph which represents the empirical functional relation among the response with the vertical axis and two factors on horizontal axes representing the coded levels of two explanatory factors, as the remaining factors being held at constant levels. The optimum values for the variables were achieved by moving along the major and minor axis of the contour. The plot was utilized to visualize how a response varied with differences in the factor [59]. Figure 2A, B, C, D depicted the CA activity and CaCO_3_ weight as a function of glucose and NaNO_2_; the surface plot was convex suggest that there are well-defined optimal variables. Additionally, as the variable ranges were suitable, the optimum lies in the design space. As depicted, the CA activity and CaCO_3_ weight increased with increasing glucose, while decreasing NaNO_2_ concentration and vice versa. Moreover, increasing variable concentrations resulted in decreasing the carbonatogenic parameters. Obviously, the contour-2D plot revealed significant antagonism interaction. On the other hand, surface plot and contour plot of calcium acetate and NaNO_2_ highlighted the mutual interaction influence on both carbonatogenic parameters as represented in Fig. 2E, F, G, H. Wherein, the carbonatogenic parameters raised with uplifting the concentrations of both variables synchronously till arrived to the highest possible level. Then, CA activity and CaCO_3_ weight started to decrease with more raising in variable values. Commonly, the contour plot of this mutual interaction display elliptical shape; expressing significant interaction [60]. Meanwhile, Fig. 2I, J, K, L represented the effect of calcium acetate and inoculum size, which declared insignificant synergetic interaction symbolizing by circular shape contour plot.

### The desirability function for prediction of the optimum conditions and model validation

The substantial aim of the statistical design of experiment focuses on attaining the maximum performance of carbonatogenic process, via both parameters of CA activity and CaCO_3_ weight, in the terms of optimum levels of examined variables, which could be obtained by desirability function. The factor settings with maximum desirability were: glucose 21, ca-acetate 18, NaNO_2_ 1.5 g/L and inoculum size (25%). As predicted, CA activity and CaCO_3_ weight assessed by 117.6048 U/mL and 1.0785 g with desirability value recorded 0.94012 and 0.82649, respectively. For verification of such prediction, the experiments were carried out in triplicates for each trial. The attained experimental values recorded 154 U/mL and 1.9 g with 1.8-fold increase comparing to the basal conditions; manifesting the good relation between the observed and predicted values.

It is noteworthy that The MICP process is influenced by many factors such as bacterial solution concentration, Ca^2+^ concentration, carbon, and nitrogen sources [61].

### Bioremediation of Zn^2+^and Cr^6+^ in MICP process

#### Minimum inhibitory concentration

The MIC values of *Rhodotorula sp*. MZ312359 aganist Zn^2+^ and Cr^6+^ recorded 100 and 800 ppm, respectively. Our data agreed that reported by Grujic [62], who found that *Rhodotorula sp.* had approximate MIC values; implying its potency in bioremediation process of considerable concentrations of metals.

### Monitoring of chemical changes during Zn^2+^and Cr^6+^ removal

**Fig. 3:**
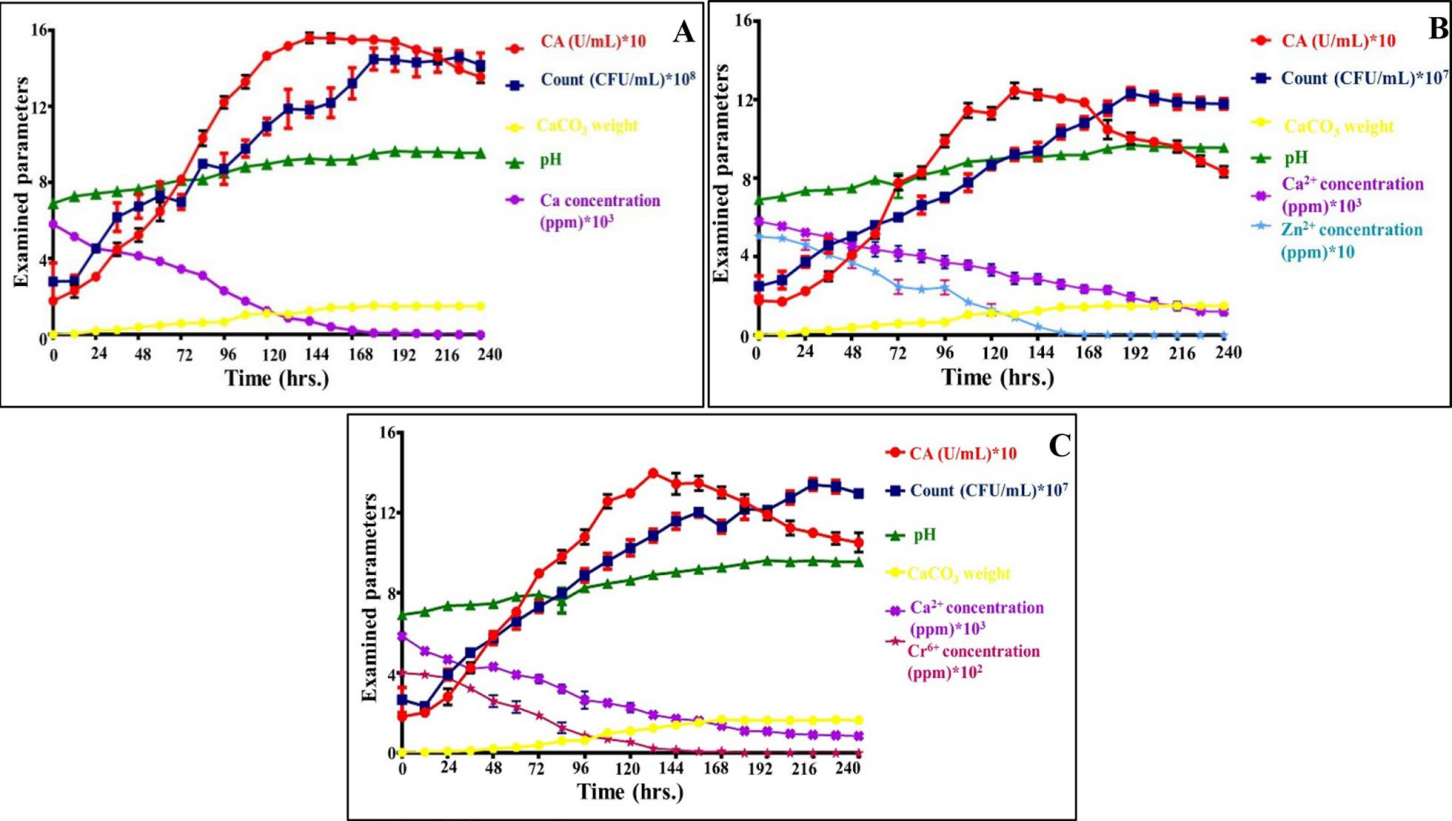
Dynamic analysis of carbonatogenesis process associated with changes in pH, cell growth, CA activity, CaCO_3_ weight and the removal of Ca^2+^, Zn^2+^ and Cr^6+^. **A** Biotic control, **B** Zn^2+^-remediation experiment, **C** Cr^6+^-remediation experiment. The results were expressed as mean ± SEM. To adjust the scale, some parameters are tripled in factor of 10 as indicated on the figure

The biomineralization dynamics of the whole remediation process was achieved through detecting changes in the chemistry of bioremediation solution in parallel to the biotic control. Therefore, the biomineralization parameters representing in CA activity, CaCO_3_ weight, pH fluctuation, soluble Ca^2+^ concentration along with microbial count, Zn^2+^ and Cr^6+^ concentrations were determined as a function of time during 10 days’ incubation (Fig. 3). Generally, a positive correlation between microbial growth, CA activity, CaCO_3_ weight was clearly evident, which was synchronized with pH uplifting and elimination of soluble (Ca^2+^, Zn^2+^ and Cr^6+^). In the biotic control, the growth profile of *Rhodotorula sp*. MZ312359 displayed a typical growth phases (lag, logarithmic and stationary) with gradual increase in the cell number and carbonatogenic parameters till reach to the maximum CA activity at 144 h, which remained stable to some extent till 180 h by recording 154.59 ± 0.193 U/mL with cell density assessed by 11.84 × 10^8^ ± 2.74 × 10^6^ CFU/mL. Besides, the solution pH recorded an elevation consistently during incubation period, which assessed by 7.1 ± 0.2 at the onset of the experiment and reached to 9.58 ± 0.015 at the end. Similarly, CaCO_3_ weight steadily increased within incubation period recorded the maximum value by 1.73 ± 0.1 g at 144 h and remained stable, without noticeable significant increase, till 10 days of incubation. Interestingly, all such increasing in the examined parameters was associated with Ca^2+^ depletion, which reached to 95.83% removal at 144 h and was entirely exhausted by the end of incubation.

Whereas, the overall diminishing in cell density concurrently with CA activity during growth stages was observed in presence of Zn^2+^ and Cr^6+^. That could be possibly attributed to the stress effect of both metals on the examined cells, which entailed acclimatization performance with them; symbolizing in slight retardation in the initial growth stages. Similar finding was reported by study performed by Mwandira et al. [63]. Broadly, in remediation experiments, the maximum CA activity displayed by 9.23 × 10^7^ ± 2.1 × 10^6^ CFU/mL and 10.88 × 10^7^ ± 2.5 × 10^6^ CFU/mL were 124.84 ± 1.24 and 140 ± 2.5 U/mL in 132 h for Zn^2+^ and Cr^6+^, respectively. This implied the leverage of both metals on the whole metabolic performance of the microbial cells. Simultaneously, a gradual raising in pH was noticed from 7.1 ± 0.2 to 9.45 ± 0.2 and 9.51 ± 0.2 for both remediated metals in the same order. On the other hand, there was vividly increasing in soluble Ca^2+^ removal percentages as inferred by ICP-OES analysis, which assessed by 51.55 and 67.05% in Zn^2+^ and Cr^6+^ remediating experiments, respectively at 144 h. Notably, such removal percentage increased upon the end of incubation and reached to 78.99 and 85.06% for Zn^2+^ and Cr^6+^, respectively. The exhaustion of such soluble form of Ca^2+^ was harmonized with its precipitation in a solid phase as determined by CaCO_3_ weight, which evaluated by 1.51 ± 0.2 and 1.62 ± 0.15 g. Interestingly, the effective metals remediation process recorded 81.9% for Zn^2+^ and 93.9% for Cr^6+^ were displayed within 144 h., respectively. A complete removal for both metals was observed at 168 h. It is worth mentioning that the absence of any precipitation in the non-biological (chemical) or abiotic control, reflects the influence of microorganisms on the change of the physical and chemical factors of the culture medium, thus, promoting the precipitation of CaCO_3_. Meanwhile, Mugwar and Harbottle [64] reported the removal of small concentration of Zn^2+^ by S*porosarcina pasteurii* through MICP within 1 week, which was in tandem with our results.

### Mineralogical and morphological analysis

While studying the dynamic of Zn^2+^ and Cr^6+^ removal, the data unveiled their absence in soluble form and implied their precipitation via MICP process. The employment of EDX, XRD, FTIR and SEM analysis emphasized their incorporation in the bioremediated deposites of CaCO_3_ crystals, confirming by such way the efficacy of *Rhodotorula sp.* MZ312359 in MICP-based bioremediation process by the dint of CA activity.

### EDX analysis

**Fig. 4:**
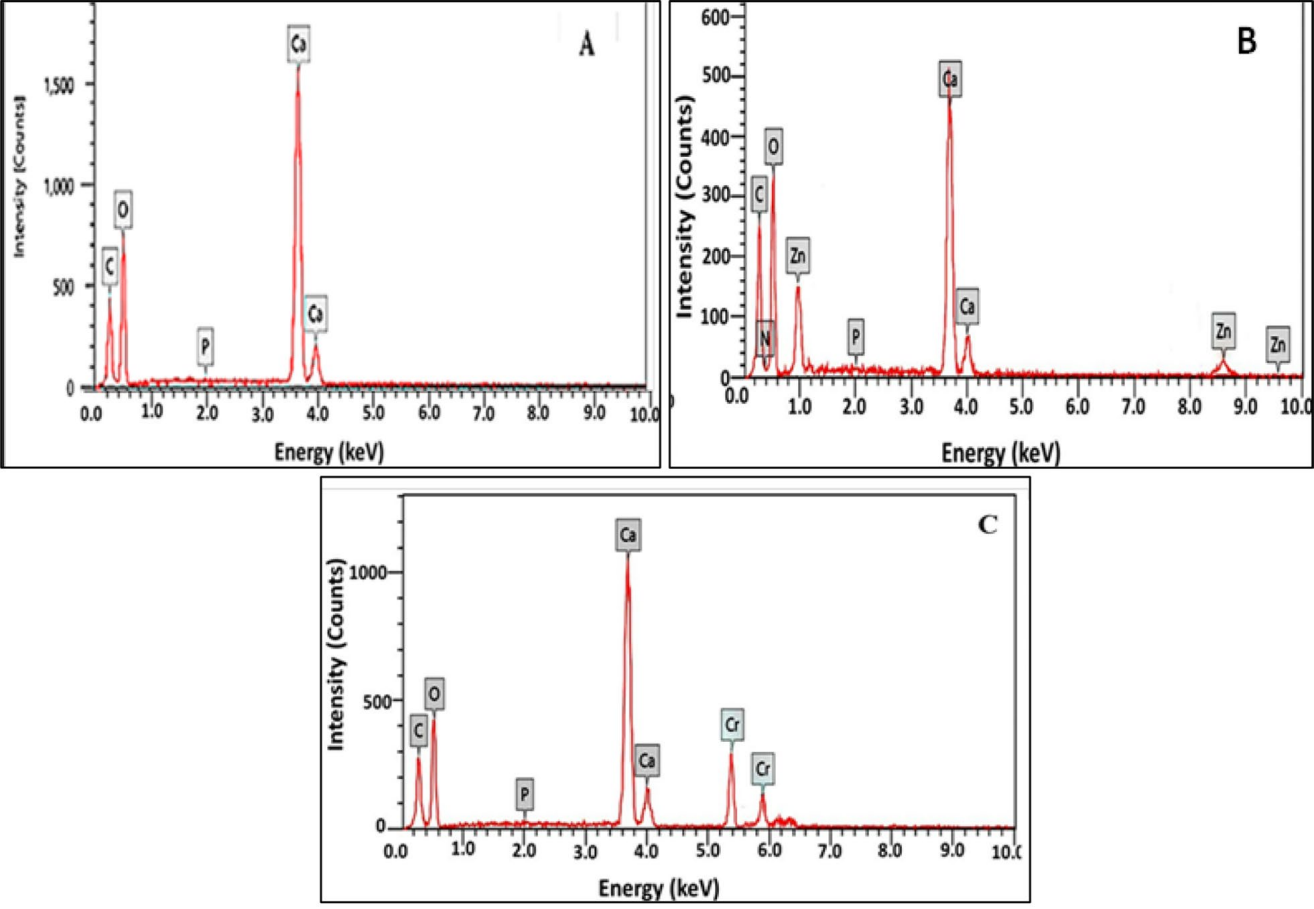
EDX pattern of biotic control (**A**) and remediated deposits containing Zn^2+^ (**B**) and Cr^6+^ (**C**)

The EDX microanalysis of the precipitated crystals in the biotic control sample expressed in Fig. 4A. The spectrum showed unique peaks at 0.277, 0.525, and 3.69 keV, which are related to the binding energy of carbon, oxygen, and calcium, sequentially [65, 66]. Other peaks relevant to the binding energy of Zn^2+^ were distinguished by the Kα and Lα characteristics at 8630 and 10.12 keV (Fig. 4B). EDX also illustrated the presence of Cr^6+^ through the distinctive emission peaks of Kα and Lα at 5.411 and 0.573, as demonstrated in Fig. 4C. These results affirmed that Zn^2+^ and Cr^6+^ were involved in calcareous bioremediated precipitates. Obviously, the existence of nitrogen (0.39 keV) and phosphorus (2.013 keV) peaks in significant amounts pointed out to the biological nature of the bioprecipitate. Actually, these elements are vital components of microbial cells biomolecules that compose proteins, nucleic acids, phospholipids and lipopolysaccharides [67]. Generally, the calcium peaks intensities and their correlating atomic percentages, which were higher than carbon peak, may reflect higher purity in structure as implied by Caicedo-Pineda et al. [68].

### Scanning electron microscope (SEM)

**Fig. 5:**
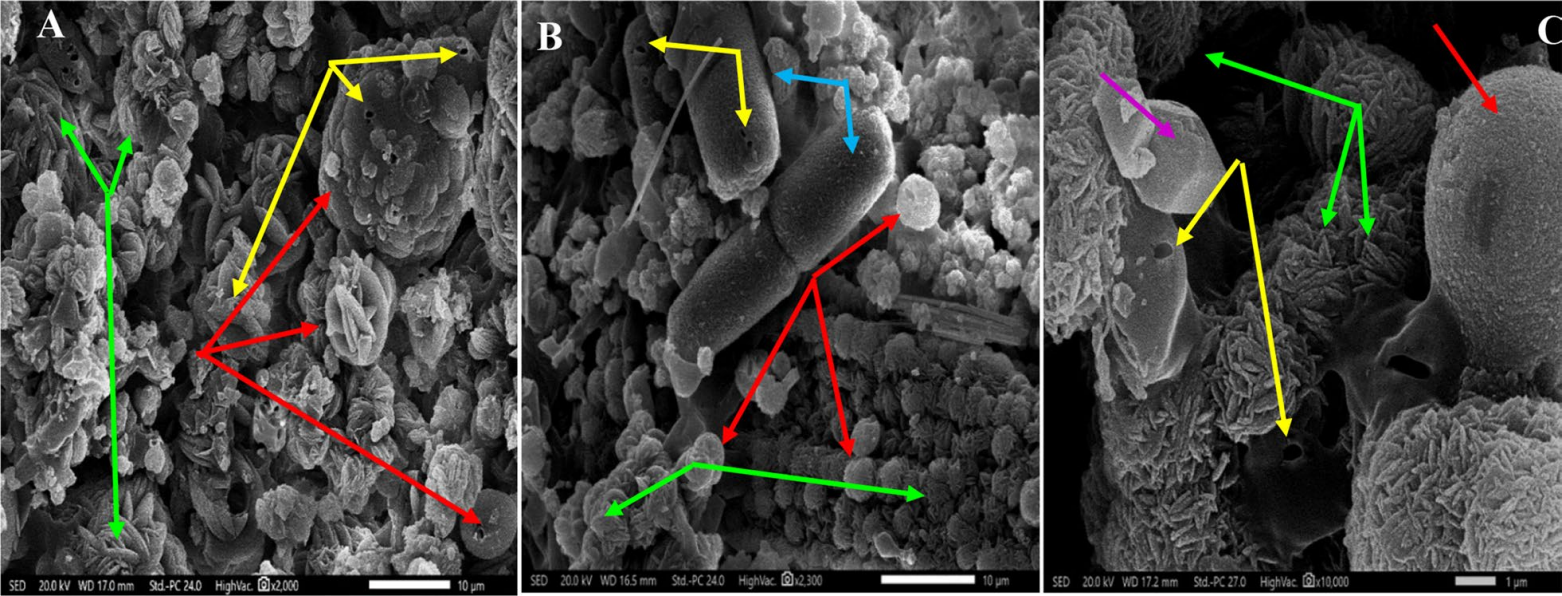
SEM micrographs of carbonate crystals; **A** Biotic control; **B** Zn^2+^ remediated sample; **C** Cr^6+^ remediated sample

The morphological and textural properties of bioremediated samples as well as the control were visualized by SEM (Fig. 5). The control micrograph illustrated acicular spheres (red arrows; 13.3 ± 2.5 µm) with other spindle shaped particles (green arrows; 1.5 ± 0.4 µm). As described by Wagterveld et al. [69], such crystal shape called morning stars. On the other hand, the bioremediated Zn^2+^ bioliths appeared mixed morphologies with spindle shaped-particles (green arrows) seemed being coalescent forming spherical particles ((red arrows; 5.3 ± 1.1 µm). Strikingly, larger cylindrical particles (blue) arrows also were detected with size assessed by 23.7 ± 3.1 µm, ornamented by holes symbolizing the fungal imprints (yellow arrows), which proposed being the initiation point or nucleus of the carbonate deposits aggregation. These structures were typical one of that found by Guo et al. [70]. Similarly, the bioremediated Cr^6+^ crystals exhibited also mixed morphologies of spindle-shaped particles forming spiked spheres (1.3 ± 0.5 µm); however, larger spherical particles with rough and wrinkled surface also were evident (5.1 ± 1.7 µm). Notably, square or cubic forms (violet arrows) were also observed with well-defined faces.

### XRD

**Fig. 6:**
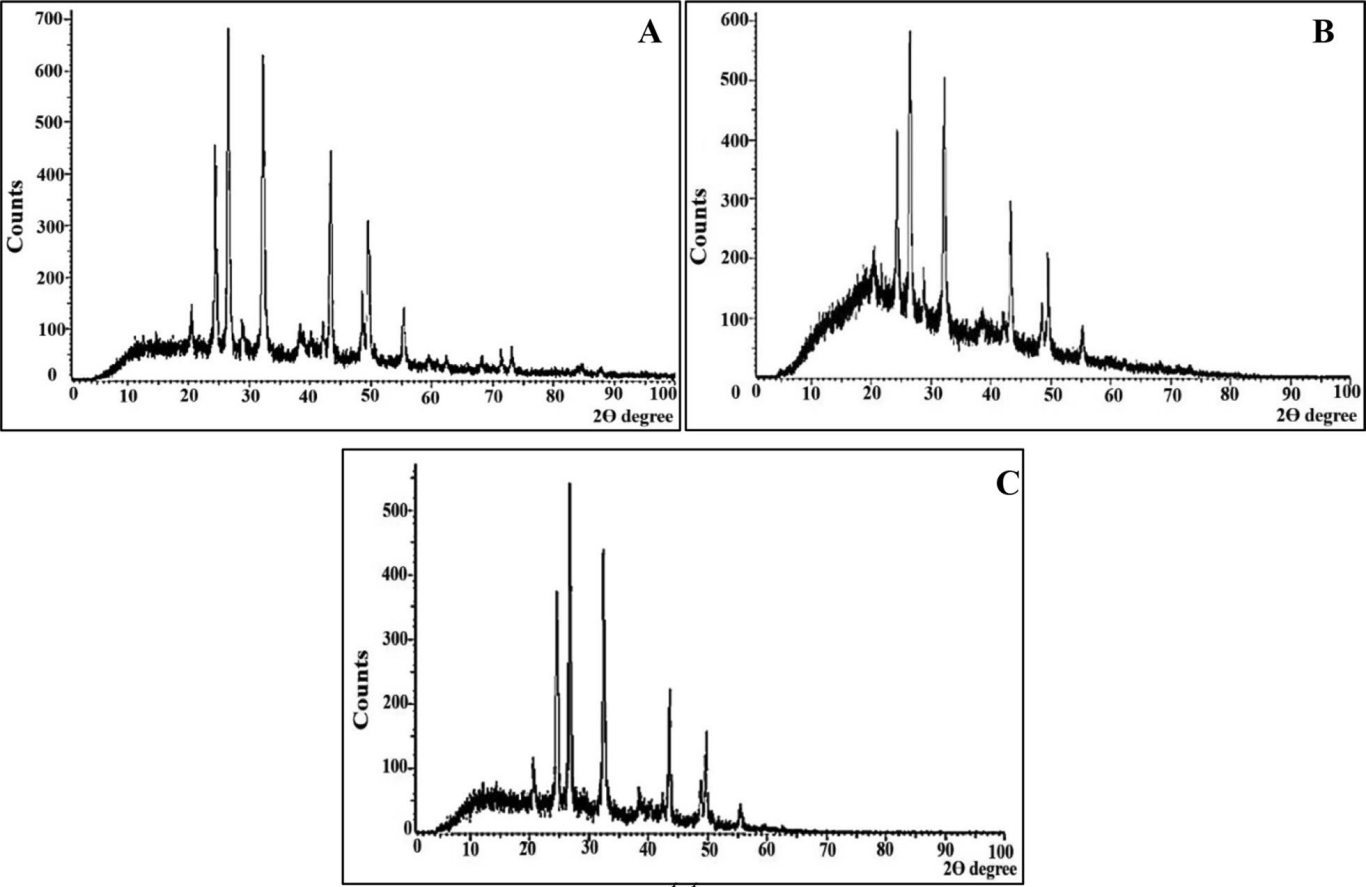
XRD Patterns of carbonate crystals; **A** Biotic control; **B** Zn^2+^ remediated sample; **C** Cr^6+^ remediated sample

The heterotrophic precipitation of CaCO_3_ is emphasized by XRD spectra. As shown in Fig. 6, sharp, distinctive, identifiable, and wide diffraction peaks were detected at 2θ values (hkl) of 20.9 (004), 24.8 (110), 27 (112), 32.8 (114), 43.7 (300), 50.1 (118), 55.9 (224) in the biotic control. These peaks accentuated the presence of crystalline CaCO_3_ deposits in vaterite phase as corroborate with the standard JCPDS card No. 72-0506 [71]. Notably, the crystallographic profile of the bioremediated samples, either Zn^2+^, or Cr^6+^, manifested the predominance of vaterite phase with minor shifting in some peaks, reduction in peaks intensities and also the disappearance of the others, comparing to the biotic control; implying the incorporation or replacement of remediated metals in vaterite matrix. Interestingly, a study conducted by Han et al. [72–74] empowered our finding. Despite the clear deposition of metal-CO_3_ (i.e., ZnCO_3_ or Cr_2_(CO_3_)_3_ was not observed in our study, their elimination process was fulfilled in stable solid phase as revealed by EDX. The obtained results might be assigned to the low crystallinity degree of both Zn^2+^or Cr^6+^ related biominerals, amorphous existence of them and the replacement of Zn^2+^/Cr^6^ to fewer positions of Ca^2+^ in the vaterite matrix. Besides, Zn^2+^/Cr^6+^ were continuously and gradual integrated/encapsulated by co-precipitation or even filled in the defect vacancy of vaterite during the uncontrolled yeast growth, which synchronize with continuous crystal nucleation/growth stages of CaCO_3_ precipitates. Subsequently, Zn^2+^/Cr^6+^ concentrations were too low to crystallize on vaterite matrix and their carbonate phases was entirely wrapped by outer CaCO_3_. Intriguingly, the flexibility of mycogenic vaterite structure with sufficient surface area, electronegativity and porosity were also taken in consideration for explaining our results. Therefore, it was plausible to describe such biogenic vaterite as disingenuous scavenger that not only absorbed Zn^2+^and Cr^6+^ but also sequestered them in robust trap. In accordance with our results [75] documented the exact results in removing Cr(VI) via chemical precipitation without obvious change in XRD pattern. Similarly, [1] supports our finding, in which all minerals precipitated chemically, under controlled conditions of Ca^2+^-binary combining with either Cd^2+^ or Zn^2+^, were only calcite peaks without any evidence of other metal-carbonate phases (i.e., otavite and smithsonite); implying the incorporation of both metals inside calcite crystal rather than adsorbed on the surface, followed by precipitating of Zn^2+^-bearing calcite and Cd^2+^-bearing calcite in lieu of end-member carbonate phase. Likewise, Hua et al. [76], highlighted that the adsorption of Cr^6+^ during chemical synthesis of CaCO_3_ crystals did not modify the mineral structure. In parallel, a study implemented by Tang et al. [77] assured the efficiency of decreasing the concentration of soluble Cr(VI) in co-precipitation process; suggesting its preferential incorporation into calcite lattice in the form of carbonate-bound Cr(VI) throughout the crystal growth process. Meanwhile, a ureolytic *Vibrio harveyi* strain selectively transformed CaCO_3_ polymorph and maintained vaterite stabilization in the presence of Zn^2+^, comparing to Pb^2+^, Cd^2+^ and Cr^6+^ [78]. In the same sense, Qiao et al. [79] found that ureolytic strains of *Lysinibacillus sp.,* and *Pseudochrobactrum sp*. removed about 48% and 53% of soluble Zn^2+^ (80 ppm) and their XRD results didn’t match any reference XRD patterns of standard zinc carbonate, zinc hydrogen, zinc oxygen or even zinc hydroxide precipitations, although a clear and distinct EDX-signal was detected. All these scholars also harmonized with our findings. Besides, Al Disi et al. [80] ascribed the ability of oil-degrading *Providencia rettgeri* and *Pseudomonas aeruginosa* in remediating Cr^6+^, Cu^2+^, Zn^2+^ and Ni^2+^ through interacting of these heavy metals with MICP-products, which were calcite (82%) and brushite (18%), whereas, their enhancing/inhibiting calcite solubility based on metals type and their content. On the contrary, *Penicillium chrysogenum* CS1 immobilized a relatively lower amount (i.e., 34%) of Cr (VI) in solution containing 200 mg through fungal-based MICP in 12 days in the form of chromium oxide carbonate as identified by XRD analysis [81]. Besides, strains of *Sporosarcina luteola* possessed the ability to precipitate Mn^2+^, Cd^2+^, Sr^2+^, Pb^2+^, Ba^+^, Zn^2+^, and Mg^+^ in their carbonates of rhodochrosite (MnCO_3_), otavite (CdCO_3_), strontianite (SrCO_3_), cerussite (PbCO_3_), witherite (BaCO_3_), hydrozincite (Zn_5_ (CO_3_)^2^ (OH)^6^) and hydromagnesite (Mg^5^(CO_3_)4 (OH)_2_·4H_2_O) [82]. Generally, the discrepancies in results among such recent studies could be attributed to the differences in microbial remediation patterns (i.e., diverse metabolic behaviors, versatile microbial response and metal selectivity strategy) toward different metals during MICP [83]. Interestingly, multiple concurrent immobilization mechanisms such as membrane surface ion exchange, adsorption, covalent binding, non-specific binding and particulate entrapment are possibly triggered along with calcite precipitation as referred by Qiao et al. [79].

### Fourier transform infrared spectroscopy (FTIR)

**Fig. 7:**
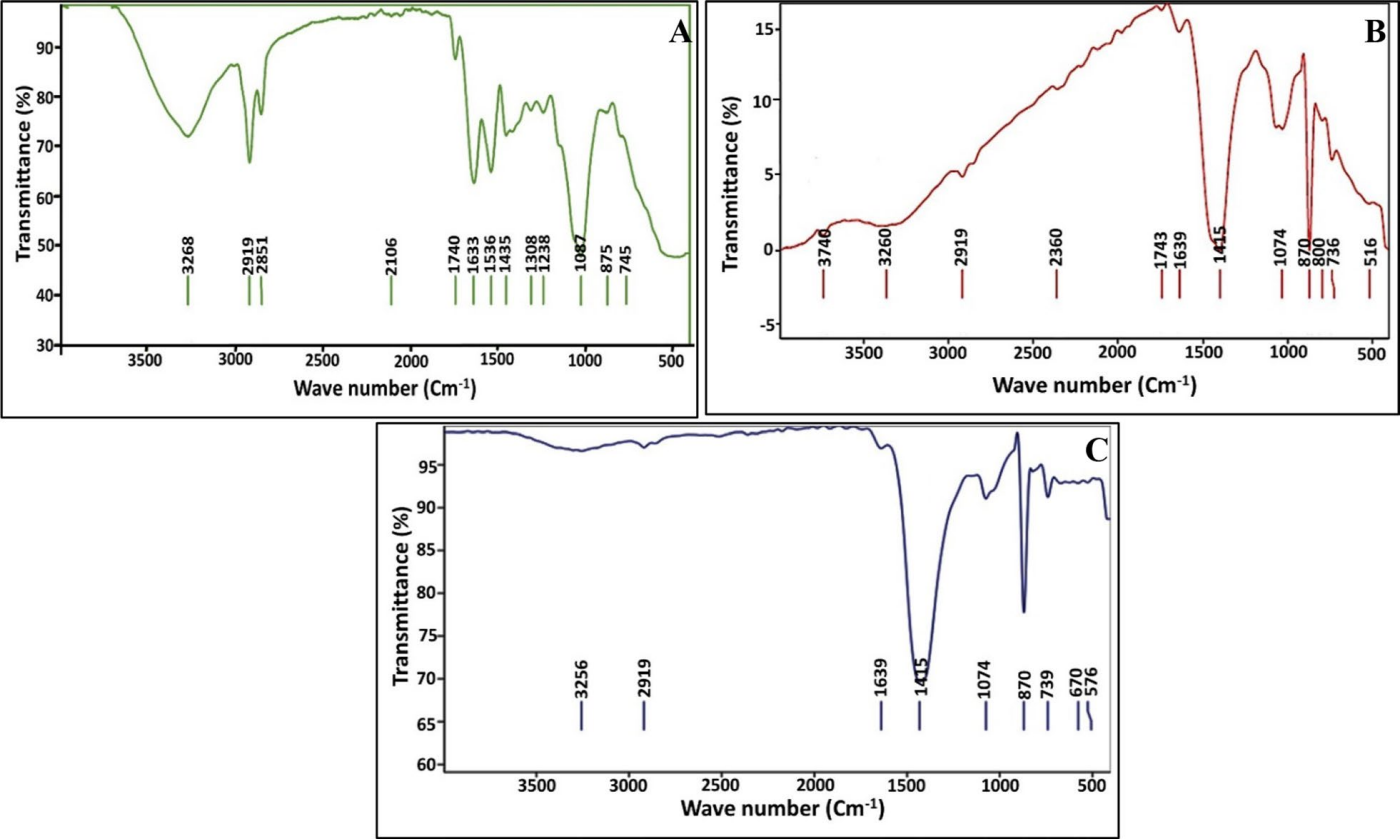
FTIR spectrum of carbonate crystals; **A** Biotic control; **B** Zn^2+^ remediated sample; **C** Cr^6+^ remediated sample

FTIR spectroscopy is an expedient tool in providing important information about the structure, functional groups associated with any examined molecule and also differentiates between different carbonate polymorphs as well. Figure 7 manifested the existence of common bands in the rejoin of 3000–3800 cm^−1^ that could be ascribed to stretching vibrations of O–H groups of the adsorbed water molecule as refereed by Kadir [84]. Besides, the vibration bands around 2357 cm^−1^ could be ascribed to carbon dioxide in the atmosphere [85]. Meanwhile, the, the vibration band related to the stretching C–H functional group was found at 2919 and 2851 cm^−1^ as referred by Hamedi et al. [86]. In addition, the peaks at the rejoin of 2396 to 2100 cm^−1^ might concern carbon dioxide in the atmosphere [85]. However, peaks at 1633 and 1639 cm^−1^ would be ascribed to amide I signature of proteins as described by Saracho et al. [87]. A same finding was recorded by Rodriguez-Navarro et al. [88], who referred to the long-term stability of CaCO_3_ polymorphs by the virtue of tight binding between amide groups and CaCO_3_ molecules. In addition, the singe for the combination of the main vibration frequencies of CO_3_^2−^ assigned between ν1, (symmetric stretching) and ν4 (in-plane bending) was implied from the spectral peak at 1743 cm^−1^ [89]. Interestingly, our results matched those obtained by Zain and Kadir [84]. Also, the characterstic peaks of vaterite were detected at IR frequencies of 745, 870, 1087 and 1435 cm^−1^, which are assigned to in-plane bending vibration (ν4), out-of plane bending modes (ν2), symmetric stretching (ν1) and asymmetric stretching vibration (ν3) of CO_3_^2−^, respectively [84]. Notably, the shifting in the typical vaterite bands in the bioremediated samples was observed; implying the incorporation of the metals, ionic bond breaking and rearrangement process inside vaterite lattice. In addition, the appearance of extra peaks at 516, 576 and 670 cm^−1^ revealed the presence of metal oxide absorption bands. Generally, any peaks at the range of 400–700 cm^−1^ highlights the existence of metals/metal oxides as stated by Hassan et al. [90]. Eventually, the tight association of fungal biomolecules (e.g., proteins, extracellular polysaccharide, glycoproteins, phospholipids, nucleic acids, etc.) with carbonate structure furnished the biominerals with higher stability and lower solubility, which in turn block the release of sequestered metals back to the remediated environment. As denoted by Li et al. [74] the biogenically synthesized CaCO_3_ crystals exhibited a potent stability and less solubility than that formed under abiotic environments.

Collectively, based on all previous data, the mechanism of Zn^2+^ and Cr^6+^ removal mediated by CA of *Rhodotorula sp*. MZ312359 in MICP process could be deduced. Initially, the growth and proliferation of fungal cell was continued with oxidation of carbon sources (i.e., glucose and acetate) under oxic conditions; generating herby CO_2_ (Equations. 8 and 9). At this time, the interconversion of CO_2_ implements by the catalysis of CA enzyme; generating dissociated ions of carbonic acid (H_2_CO_3_), which produces bicarbonate (HCO_3_^−^) (Eq. 10) [91]. This continuous process would in turn elevate pH that eventually favors CaCO_3_ precipitation in the presence of Ca^2+^ ions through three-stage process of nucleation, growth and crystallization. Wherein, the fungal cells themselves serving as nucleation sites by the dint of their electronegativity nature. The negatively charged macromolecules such as polysaccharide, glycoproteins, lipids, proteins and lipopolysaccharide contain functional groups (e.g., carboxylic (R-COO^−^), sulfonate (R-SO_3_), and phosphate (R-PO_4_^2−^), etc.) that attract the cations via attractive van der Waals forces [92]. Once the nuclei of CaCO_3_ precipitated on the fungal cell, in a supersaturated solution, crystal development begins via atom-by-atom addition during the second stage of crystal growth; generating larger size particles of CaCO_3_ deposits (Eqs. 11-13). Upon continuous fungal metabolic activity, which was synchronized with the production of CO_3_^−^ and Ca^2+^ions and pH raising, the maturation of CaCO_3_ crystals executed in the third crystallization stage.

8 $${\text{C}}_{6}{\text{H}}_{12}{\text{O}}_{6} (\text{s}) + 6 {\text{O}}_{2} (\text{g}) \to 6\text{ C}{\text{O}}_{2} (\text{g}) + 6 {\text{H}}_{2}\text{O}$$

9 $${({\text{CH}}_{3}\text{COO})}_{2}\text{Ca }(\text{s}) + 4{\text{O}}_{2} (\text{g}) \to 4{\text{CO}}_{2} (\text{g}) + 3{\text{H}}_{2}\text{O }+\text{ CaO}$$

10 $${\text{CO}}_{2} + {\text{H}}_{2}\text{O }\leftrightarrow {\text{H}}_{2}{\text{CO}}_{3}\leftrightarrow {{\text{HCO}}_{3}}^{-} + {\text{H}}^{+}$$

11 $$\text{HC}{{\text{O}}_{3}}^{-} \leftrightarrow {\text{H}}^{+} +\text{ C}{{\text{O}}_{3}}^{2-}$$

12 $${\text{Ca}}^{2+} + {\text{cell}}^{-} \to \text{ Cell }- {\text{Ca}}^{2+}$$

13 $$\text{Cell }- {\text{Ca}}^{2}+ + {{\text{CO}}_{3}}^{2-} \to \text{ Cell }-\text{ CaC}{\text{O}}_{3} \downarrow$$

In the concern of bioremediation process, it was accomplished within a proper time frame; producing stable solid phase, in spite of the absence of metal in the form of metal- CO_3_ phase. Wherein, two suggestions governed the MICP- process driven by the examined fungus of the current study. The first one assumes both metals were remediated through the substitution of Ca^2+^ with Zn^2+^ and Cr^6+^, in particular with the presence of unconsumed percentage of soluble Ca^2+^ that assessed by 21.01 and 14.94% for Zn^2+^ and Cr^6+^, respectively, comparing to the biotic control that precipitated the soluble Ca^2+^ entirely till the end of remediation experiment. In a convergent finding, Eltarahony [40] reported that *Proteus mirabilis* 10B eliminated Pb^2+^ and Hg^2+^ through Ca^2+^ substitution in MICP process mediated by nitrate reductase enzyme. It is plausible to state that ionic exchange process between cations is controlled by several parameters for instance ionic radius, electronegativity, metals hydrolysis constant, ionic radius, electronegativity, atomic radius and hydrated radius [25]. Remarkably, Zhu and Dittrich [25], Chada et al. [93] revealed that the divalent cations (Pb^2+^, Cd^2+^, Sr^2+^, Co^2+^ and Zn^2+^) have ionic radii that are closed to Ca^2+^, which subsequently enable their replacement and inclusion in calcite crystals. In the same extent, the removal of toxic Pb^2+^ and Cr^6+^ by *Penicillium chrysogenum* in MICP was achieved proposing their replacement with CO_3_^−^ anion in calcite lattice [81].

The another suggestion depends on the characterstic properties of biogenic vaterite including its flexible structure with considerable surface area and sufficient porosity, which in turn boosted the incorporation of Zn^2+^ and Cr^6+^ inside vaterite lattice. Notable, [81] recorded the same observation. However, the electronegativity of vaterite also facilitated the gradual capturing of Zn^2+^ and Cr^6+^during the development and growth stages of vaterite crystals; signifying the absorptive with sequestering capacity of vaterite. Interestingly, [75] found the same feature during the removal of Cr^6+^ by chemical precipitation process. In this regard, Sdiri and Higashi [94] manifested the effective utilization of natural limestones in eliminating 10 ppm of Pb^2+^ within 6 h. In the same extend, Du et al*.* [95], employed mollusk shells that mainly composed of calcite and aragonite as an efficient biosorbent for remediating divalent Pb, Cd and Zn. Broadly, all these scholars ascertained the biosorption capacity of CaCO_3_, unveiling the promising and potent performance of either MICP process or even its byproduct in purifying contaminated ecosystem.

Ultimately, the mineralogical characterization techniques assured the engulfment of Zn^2+^/Cr^6+^in vaterite co-precipitated remediated products as possible action of carbonic anhydrase. Moreover, they reflected not only the scavenging role of CaCO_3_ in chelating soluble heavy metals but also their stabilization inside potent trap, which displayed superior efficiency than adsorption [93]. Arguably, the adsorption is superficial process and based mainly on bonding specifications between adsorbents and adsorbate, which substantially depends on solution ionic strength, pH and availability of sufficient functional groups [96]. Strikingly, the precipitation approach in hydroxides forms was extensively and efficiently studied in heavy metals removal, nevertheless, the tendency to form soluble anionic hydroxyl complexes through water redissolution considered being the main drawback [97, 98]. In general, the remediation of heavy metals through MICP deemed as effective, easy, ecofriendly and inexpensive procedure to restrict toxic metals mobility, bioavailability and their release to the surrounding milieu with facilitated separation and without additional step like coagulation, filtration, or flocculation.

## Conclusion

In conclusion, the current study dedicated on maximizing carbonatogenic process, which was implemented by the catalysis of CA enzyme of *Rhodotorula sp*. MZ 312359 via employing statitical experimental designs (i.e., PBD and CCD) to remeidate Zn^2+^ and Cr^6+^ in MICP, for the first time. Such statistical approaches enhanced carbonatogenic parameters of CA activity and also CaCO_3_ weight by 1.8-fold, comparing to un-optimized basal media. Under the optimized precipitating conditions, *Rhodotorula sp*. MZ 312359 eliminated entirely 50 and 400 ppm of Zn^2+^ and Cr^6+^, respectively within 7 days of incubation. Subsequently, the mineralogical analysis including EDX, SEM, FTIR and XRD confirmed the immobilization of soluble toxic metals inside a potent trap of vaterite lattice, which deemed as a promising strategy for cost-effective, environmentally benign, sustainable and effective mean for alleviation heavy metals toxicity.

## Data Availability

All data generated or analyzed during this study are included in this article. No datasets were generated or analysed during the current study https://www.ncbi.nlm.nih.gov/nuccore/MZ312359.1/.

## Abbreviations

MICP: Microbial induced calcium carbonate precipitation
CaCO_3_: Calcium carbonate
WHO: World Health Organization
CA: Carbonic anhydrase
SRB: Sulphate reducing bacteria
EDX: Energy dispersive X-ray spectrometry
SEM: Scanning electron microscopy
XRD: X-ray diffraction
FTIR: Fourier transform infrared spectroscopy
CO_2_: Carbon dioxide
Cr: Chromium
Zn: Zinc
ANOVA: Analysis of variance

## References

[1] Kim J, Lee S, Fenter P, Myneni S, Nikitin V, Peters C. Carbonate coprecipitation for Cd and Zn treatment and evaluation of heavy metal stability under acidic conditions. Environ Sci Technol. 2023;57:3104–13. 36781166 10.1021/acs.est.2c07678 PMC9979612

[2] Medina-Armijo C, Isola D, Illa J, Puerta A, Vinas M, Prenafeta-Boldú FX. The Metallotolerance and Biosorption of As(V) and Cr(VI) by Black Fungi. J fungi. 2024;10:47. 10.3390/jof10010047 PMC10817489 38248956

[3] Vignati D, Janusz D, Mamadou Beye L, Maurizio P, Ferrari JD. Chromium(VI) is more toxic than chromium(III) to freshwater algae: a paradigm to revise. Ecotoxicol Environm Safety. 2010;73(5):743–9. 10.1016/j.ecoenv.2010.01.011 20138363

[4] Thomas M, Melichová Z, Šuránek M, Kuc J, Wieckol-Ryk A, Lochy’nski P. Removal of zinc from concentrated Galvanic wastewater by Sodium trithiocarbonate: process optimization and toxicity assessment. Molecules. 2023;28:546. 36677604 10.3390/molecules28020546 PMC9860917

[5] Zhang F, Du N, Li H, Shue S, Wanguo H. Sorbent effect on the sorption of Cr(VI) on a Mg6AlFe-layered double hydroxide and its calcined product in aqueous solutions. Colloid Polym Sci. 2015;293(7):1961–9. 10.1007/s00396-015-3592-x

[6] Rajendran S, Priya TA, Khoo KS, Hoang TK, Ng HS, Munawaroh HS, Karaman C, Orooji Y, Show PL. A critical review on various remediation approaches for heavy metal contaminants removal from contaminated soils. Chemosphere. 2022;1(287):132369. 10.1016/j.chemosphere.2021.132369 34582930

[7] Li Q, Zhang M, Wei B, Lan W, Wang Q, Chen Z, Zhao H, Liu D, Gadd MG. Fungal biomineralization of toxic metals accelerates organic pollutant removal. Cuur Bio. 2024;34(10):2077–84. 10.1016/j.cub.2024.04.005 38663397

[8] Anendita H, Meera Y. Biomineralization of carbon dioxide by carbonic anhydrase. Biocatalys Agricul Biotechnol. 2023;51:102–755.

[9] Dyer T. Influence of cement type on resistance to attack from two carboxylic acids. Cem Concr Compos. 2017;83:20–35. 10.1016/j.cemconcomp.2017.07.004

[10] Alori ET, Gabasawa AI, Elenwo CE, Agbeyegbe OO. Bioremediation techniques as affected by limiting factors in soil environment. Front Soil Sci. 2022;4(2):937186. 10.3389/fsoil.2022.937186

[11] Li T, Zhang H, Tan X, Zhang R, Wu F, Yu Z, Su B. New insights into Saccharomyces cerevisiae induced calcium carbonate precipitation. Front Bioeng Biotechnol. 2023;11:1261205. 37720316 10.3389/fbioe.2023.1261205 PMC10500597

[12] Ma Y, Prasad M, Rajkumar M, Freitas H. Plant growth promoting rhizobacteria and endophytes accelerate phytoremediation of metalliferous soils. Biotechnol Adv. 2011;29:248–58. 21147211 10.1016/j.biotechadv.2010.12.001

[13] Bai Y, Guo XJ, Li YZ, Huang T. Experimental and visual research on the microbial induced carbonate precipitation by *Pseudomonas aeruginosa*. AMB Express. 2017;47:8–5. 10.1186/s13568-017-0358-5 PMC5342990 28275994

[14] Liu P, Cheng Y, Chen L, Shao GH. Application of microbial mineralization in the treatment of sintering red mud. J Cent South Univ. 2023;30(9):3057–68. 10.1007/s11771-023-5381-x

[15] Gilmour KA, Ghimire PS, Wright J, Haystead J, Dade-Robertson M, Zhang M, James P. Microbially induced calcium carbonate precipitation through CO_2_ sequestration via an engineered Bacillus subtilis. Micro Cell Fact. 2024;23:168. 10.1186/s12934-024-02437-7 PMC11163794 38858761

[16] Eltarahony M, Kamal A, Zaki S, Abd-El-Haleem D. Heavy metals bioremediation and water softening using ureolytic strains Metschnikowia pulcherrima and Raoultella planticola. J Chemi Tech Biotech. 2021;96(11):3152–65. 10.1002/jctb.6868

[17] Wilcox SM, Mulligan CN, Neculita CM. Microbially induced calcium carbonate precipitation as a bioremediation technique for mining waste. Toxics. 2024;12(2):107. 38393202 10.3390/toxics12020107 PMC10891697

[18] Zúñiga-Barra H, Ostojic C, Torres-Aravena Á, Rivas M, Vílchez C, Jeison D. Use of photosynthetic MICP to induce calcium carbonate precipitation: prospecting the role of the microorganism in the formation of CaCO3 crystals. Algal Res. 2024;1(80):103499. 10.1016/j.algal.2024.103499

[19] Lin Y, Turchyn A, Steiner Z, Bots P, Lampronti G, Tosca N. The role of microbial sulfate reduction in calcium carbonate polymorph selection. Geochim Cosmochim Acta. 2018;237:184–204. 10.1016/j.gca.2018.06.019

[20] Konstantinou C, Wang Y. Unlocking the potential of microbially induced calcium carbonate precipitation (MICP) for hydrological applications: a review of opportunities, challenges, and environmental considerations. Hydrology. 2023;10(9):178. 10.3390/hydrology10090178

[21] Abdelsamad R, Al Disi Z, Abu-Dieyeh MA, Al-Ghouti M, Zouari N. Evidencing the role of carbonic anhydrase in the formation of carbonate minerals by bacterial strains isolated from extreme environments in Qatar. Heliyon. 2022;8(10): e11151. 36311368 10.1016/j.heliyon.2022.e11151 PMC9614864

[22] Xiao L, Lian B. Heterologously expressed carbonic anhydrase from Bacillus mucilaginosus promoting CaCO3 formation capturing atmospheric. Carbo Evap. 2016;31:39–45. 10.1007/s13146-015-0239-4

[23] He Y, Duan W, Xue B, Cong X, Sun P, Hou X, Liang YK. Os_CA1 affects photosynthesis, yield potential, and water use efficiency in rice. Int J Mol Sci. 2023;24:5560. 36982632 10.3390/ijms24065560 PMC10056782

[24] Bose H, Satyanarayana T. Microbial carbonic anhydrases in biomimetic carbon sequestration for mitigating global warming: prospects and perspectives. Front Microbiol. 2017;8:1615. 28890712 10.3389/fmicb.2017.01615 PMC5574912

[25] Zhu T, Dittrich M. Carbonate precipitation through microbial activities in natural environment and their potential in biotechnology: a review. Bioeng Biotechnol. 2016;4:1–21. 10.3389/fbioe.2016.00004 PMC4718973 26835451

[26] Nicula NO, Lungulescu EM, Rîmbu GA, Marinescu V, Corbu VM, Csutak O. Bioremediation of wastewater using yeast strains: an assessment of contaminant removal efficiency. Int J Environ Res Public Health. 2023;8(6):4795. 10.3390/ijerph20064795 PMC10048942 36981703

[27] Zhang J, Keasling JD, Avalos J. Engineered yeast brews precursors of anticancer drug vinblastine. Nature. 2022;609:341. 36045165 10.1038/s41586-022-05157-3

[28] Shahat AS. Antioxidant and anticancer activities of yeast grown on commercial media. Inter J Biol Chem Sci. 2017;11(5):2442–55. 10.4314/ijbcs.v11i5.39

[29] Pang Y, Zhang H, Wen H, Wan H, Wu H, Chen Y, Li S, Zhang L, Sun X, Li B, Liu X. Yeast probiotic and yeast products in enhancing livestock feeds utilization and performance: an overview. J Fungi. 2022;11(11):1191. 10.3390/jof8111191 PMC9695268 36422012

[30] Raita S, Kusnere Z, Spalvins K, Blumberga D. Optimization of yeast cultivation factors for improved SCP production. Environ Clim Technol. 2022;26(1):848–61. 10.2478/rtuect-2022-0064

[31] Elsayis A, Hassan SW, Ghanem KM, Khairy H. Optimization of melanin pigment production from the halotolerant black yeast Hortaea werneckii AS1 isolated from solar salter in Alexandria. BMC Microbiol. 2022;8(1):92. 10.1186/s12866-022-02505-1 PMC8991569 35395716

[32] Nuanpeng S, Thanonkeo S, Klanrit P, Yamada M, Thanonkeo P. Optimization conditions for ethanol production from sweet sorghum juice by thermotolerant yeast Saccharomyces cerevisiae: using a statistical experimental design. Fermen. 2023;9(5):450. 10.3390/fermentation9050450

[33] Melo NT, Oliveira Junqueira AC, Lima LF, Oliveira KB, Dos Reis MC, Franco OL, Paes HC. Just around the corner: advances in the optimization of yeasts and filamentous fungi for lactic acid production. J Fungi. 2024;9(3):207. 10.3390/jof10030207 PMC10971269 38535215

[34] El-Fakharany EM, Abu-Serie MM, Ibrahim A, Eltarahony M. Anticancer activity of lactoferrin-coated biosynthesized selenium nanoparticles for combating different human cancer cells via mediating apoptotic effects. Sci Rep. 2023;13(1):9579. 37311791 10.1038/s41598-023-36492-8 PMC10264462

[35] Almahdy AG, El-Sayed A, Eltarahony M. A novel functionalized CuTi hybrid nanocomposites: facile one-pot mycosynthesis, characterization, antimicrobial, antibiofilm, antifouling and wastewater disinfection performance. Microb Cell Factor. 2024;23(1):148. 10.1186/s12934-024-02400-6 PMC11112895 38783243

[36] Saleh H, Abdelrazak A, Elsayed A. Optimizing production of a biopesticide protectant by black yeast. Egypt J Biol Pest Control. 2018;28:72. 10.1186/s41938-018-0078-4

[37] Freimoser FM, Rueda-Mejia MP, Tilocca B, Migheli Q. Biocontrol yeasts: mechanisms and applications. W J Microbiol Botechnol. 2019;35(10):154. 10.1007/s11274-019-2728-4 PMC6773674 31576429

[38] Kowalska J, Krzymińska J, Tyburski J. Yeasts as a potential biological agent in plant disease protection and yield improvement—A short review. Agricul. 2022;6(9):1404. 10.3390/agriculture12091404

[39] El-Bestawy EA, El-Batouti MM, Zabermawi NM, Zaghlol HM. Removal of heavy metals, turbidity and coliform from contaminated raw drinking water using Saccharomyces cerevisiae, the Baker’s yeast. Sust Chem Pharm. 2023;1(33):101131.

[40] Eltarahony M, Zaki S, Abd-El-Haleem D. Aerobic and anaerobic removal of lead and mercury via calcium carbonate precipitation mediated by statistically optimized nitrate reductases. Sci Rep. 2020;10(1):1–20. 32132620 10.1038/s41598-020-60951-1 PMC7055279

[41] Barbero R, Carnelli L, Simon A, Kao A, Monforte A, Ricco M, Bianchi D, Belcher A. Engineered yeast for enhanced CO_2_ mineralization. Energy Environ Sci. 2013;6:660–74. 25289021 10.1039/c2ee24060b PMC4185198

[42] Eltarahony M, Zaki S, Kamal A, Abd-El-Haleem D. Calcite and vaterite biosynthesis by nitrate dissimilating bacteria in carbonatogenesis process under aerobic and anaerobic conditions. Geomicrobiol J. 2021;38(9):791–808. 10.1080/01490451.2021.1951398

[43] Nunes P, Demaurex N, Dinauer M. Regulation of the NADPH oxidase and associated ion fluxes during phagocytosis. Traffic. 2013;14:1118–31. 23980663 10.1111/tra.12115

[44] Kumar A, Singhal K, Sharma R, Vyas G, Kumar V. Molecular characterization of *Catharanthus Roseus* cultivars from various regions of rajasthan based on rapd marker. Intern J Pharma Sci Res. 2014;5(9):3936–41.

[45] El-Shall H, Abu-Serie M, Abu-Elreesh G, Eltarahony M. Unveiling the anticancer potentiality of single cell oils produced by marine oleaginous *Paradendryphiella sp*. under optimized economic growth conditions. Sci Rep. 2023;13:20773. 38008815 10.1038/s41598-023-47656-x PMC10679151

[46] Vashisht R, Attri S, Sharma D, Shukla A, Goel G. Monitoring biocalcification potential of *Lysinibacillus sp*. isolated from alluvial soils for improved compressive strength of concrete. Microbiol Res. 2018;207:226–31. 29458858 10.1016/j.micres.2017.12.010

[47] Sharma T, Sharma S, Kamyab H, Kumar A. Energizing the CO2 utilization by chemo-enzymatic approaches and potentiality of carbonic anhydrases: a review. J Clean Prod. 2020;247:119138. 10.1016/j.jclepro.2019.119138

[48] Sharma A, Bhattacharya A, Singh S. Purification and characterization of a carbonic anhydrase from *Pseudomonas fragi*. Process Biochem. 2009;44:1293–7. 10.1016/j.procbio.2009.07.022

[49] Mussagy U, Helena Ribeiro F, Jorge F, Pereir B. *Rhodotorula sp*. as a cell factory for production of valuable biomolecules. Adv Appli Microbiol. 2023;123:133–56. 10.1016/bs.aambs.2023.04.001 37400173

[50] Pacia M, Turnau K, Baranska M, Kaczor A. Interplay between carotenoids, hemoproteins and the “life band” origin studied in live *Rhodotorula mucilaginosa* cells by means of Raman microimaging. Analyst. 2015;140(6):1809–13. 25654139 10.1039/C4AN01787K

[51] Allahkarami S, Akhavan Sepahi A, Hosseini H, Razavi M. Isolation and identification of carotenoid-producing *Rhodotorula sp*. from Pinaceae forest ecosystems and optimization of in vitro carotenoid production. Biotechnol Rep. 2021;32: e00687. 10.1016/j.btre.2021.e00687 PMC8593566 34815952

[52] Senthilkumar N, Tamizharasan T, Gobikannan S. Application of response surface methodology and firefly algorithm for optimizing multiple responses in turning AISI 1045 steel. Arab J Sci Engin. 2014;39(11):8015–30. 10.1007/s13369-014-1320-3

[53] Phoa F, Wong W, Xu H. The need of considering the interactions in the analysis of screening designs. A J Chemometr Soci. 2009;23(10):545–53. 10.1002/cem.1252

[54] Reddy M, Rao C, Lakshmana S. Evaluation of process parameters and media components by Plackett-Burman design for enhancement of biomass using *cyanobacteria* (Anabaena ambigua). Intern J Chem Tech Res. 2012;4(2):761–6.

[55] Mojtaba A, Fardin K. Optimization of enzymatic extraction of oil from *Pistacia Khinjuk* seeds by using central composite design. Food Sci Technol. 2013;1(3):37–43. 10.13189/fst.2013.010301

[56] Anuar N, Mohd A, Saat N, Aziz N, Mat TR. Optimization of extraction parameters by using response surface methodology, purification, and identification of anthocyanin pigments in *Melastoma malabathricum* fruit. Scien W J. 2013;2013:810547. 10.1155/2013/810547 PMC3794562 24174918

[57] Taavitsainen V. Experimental optimization and response surfaces. Chemomet Pract Appl. 2012;91:138.

[58] Dutka M, Ditaranto M, Løvås T. Application of a central composite design for the study of NOx emission performance of a low NOx burner. Energies. 2015;8(5):3606–27. 10.3390/en8053606

[59] El-Naggar N, Abdelwahed N. Application of statistical experimental design for optimization of silver nanoparticles biosynthesis by a nanofactory *Streptomyces viridochromogenes*. J Microbiol. 2014;52(1):53–63. 24390838 10.1007/s12275-014-3410-z

[60] De Lima C, Coelho L, Contiero J. The use of response surface methodology in optimization of lactic acid production focus on medium supplementation, temperature and pH control. Food Technol Biotechnol. 2010;48(2):175–81.

[61] Liu Y, Ali A, Su J, Li K, Hu R, Wang Z. Microbial-induced calcium carbonate precipitation: Influencing factors, nucleation pathways, and application in waste water remediation. Sci Total Environ. 2023;860:160439. 36574549 10.1016/j.scitotenv.2022.160439

[62] Grujic S, Vasic S, Radojevic I, Comic L, Ostojic A. Comparison of the *Rhodotorula mucilaginosa* biofilm and planktonic culture on heavy metal susceptibility and removal potential. Water Air Soil Pollut. 2017;228(2):8. 10.1007/s11270-017-3259-y

[63] Mwandira W, Nakashimab K, Kawasaki S. Bioremediation of lead contaminated mine waste by *Pararhodobacter sp*. based on the microbially induced calcium carbonate precipitation technique and its effects on strength of coarse- and fine-grained sand. Ecol Eng. 2017;109:57–64. 10.1016/j.ecoleng.2017.09.011

[64] Mugwar A, Harbottle M. Toxicity effects on metal sequestration by microbially-induced carbonate precipitation. J hazard Mater. 2016;314:237–48. 27136729 10.1016/j.jhazmat.2016.04.039

[65] Newbury D. Mistakes encountered during automatic peak identification of minor and trace constituents in electron excited energy dispersive X-ray microanalysis. Scanning J Scann Micros. 2009;31(3):91–101. 10.1002/sca.20151 19533682

[66] François F, Lombard C, Guigner J, Soreau P, Brian-Jaisson F, Martino G, Rebuffat S. Isolation and characterization of environmental bacteria capable of extracellular biosorption of mercury. Appl Environ Microbial. 2012;78(4):1097–106. 10.1128/AEM.06522-11 PMC3273009 22156431

[67] Zaki S, Eltarahony M, Abd-El-Haleem D. Disinfection of water and wastewater by biosynthesized magnetite and zerovalent iron nanoparticles via NAP-NAR enzymes of *Proteus mirabilis* 10B. Environ Sci Poll Res. 2019;26(23):23661–78. 10.1007/s11356-019-05479-2 31201708

[68] Caicedo-Pineda G, Prada-Fonseca M, Casas-Botero A, Martínez-Tejada H. Effect of the tryptone concentration on the calcium carbonate biomineralization mediated by *Bacillus cereus*. Dyna. 2018;85(205):69–75. 10.15446/dyna.v85n205.60637

[69] Wagterveld R, Yu M, Miedema H, Witkamp G. Polymorphic change from vaterite to aragonite under influence of sulfate: the “morning star” habit. J Cryst Growth. 2014;387:29–35. 10.1016/j.jcrysgro.2013.10.044

[70] Ma X, Li L, Yang L, Su C, Guo Y, Jiang K. Preparation of highly ordered hierarchical CaCO_3_ hemisphere and the application as pH value-sensitive anticancer drug carrier. Mater Lett. 2011;65:3176–9. 10.1016/j.matlet.2011.07.009

[71] Chong K, Chia C, Zakaria S, Sajab M. Vaterite calcium carbonate for the adsorption of Congo red from aqueous solutions. J Environ Chem Eng. 2014;2(4):2156–61. 10.1016/j.jece.2014.09.017

[72] Han Z, Yu W, Zhao Y, Tucker M, Yan H. The significant role of different magnesium: carbonate minerals induced by moderate halophile *Staphylococcus epidermis* Y2. Miner. 2018;8:594.

[73] Sheng M, Peng D, Luo S, Ni T, Luo H, Zhang R, Wen Y, Xu H. Micro-dynamic process of cadmium removal by microbial induced carbonate precipitation. Environ Poll. 2022;308:119585. 10.1016/j.envpol.2022.119585 35728693

[74] Li X, Wang Y, Tang J, Li K. Removal behavior of heavy metals from aqueous solutions via microbially induced carbonate precipitation driven by acclimatized *sporosarcina pasteurii*. Appl Sci. 2022;12(19):9958. 10.3390/app12199958

[75] Sun J, Zhu WT, Huang J. Characterization of primary precipitate composition formed during co-removal of W Cr (VI) with Cu (II) in synthetic wastewater. Environ Sci Poll Res. 2006;13(6):379–85. 10.1065/espr2005.10.286 17120827

[76] Hua B, Deng B, Thornton E, Yang J, Amonette J. Incorporation of chromate into calcium carbonate structure during coprecipitation. Water Air Soil Pollu. 2007;179:381–90. 10.1007/s11270-006-9242-7

[77] Tang Y, Elzinga E, Lee J, Reeder R. Coprecipitation of chromate with calcite: batch experiments and X-ray absorption spectroscopy. Geochim Cosmochim Acta. 2007;71:1480–93. 10.1016/j.gca.2006.12.010

[78] Jarwar M, Del Buey P, Sanz-Montero M, Dumontet S, Chianese E, Pasquale V. Co-Precipitation of Cd, Cr, Pb, Zn, and carbonates using *Vibrio harveyi* strain isolated from mediterranean sea sediment. Minerals. 2023;13(5):627. 10.3390/min13050627

[79] Qiao S, Zeng G, Wang X, Dai C, Sheng M, Chen Q, Xu F, Xu H. Multiple heavy metals immobilization based on microbially induced carbonate precipitation by ureolytic bacteria and the precipitation patterns exploration. Chemosph. 2021;274:129661. 10.1016/j.chemosphere.2021.129661 33979921

[80] Al Disi Z, Attia E, Ahmad M, Zouari N. Immobilization of heavy metals by microbially induced carbonate precipitation using hydrocarbon-degrading ureolytic bacteria. Biotechnol Rep. 2022;35: e00747. 10.1016/j.btre.2022.e00747 PMC9218142 35755319

[81] Qian X, Fang C, Huang M, Achal V. Characterization of fungal-mediated carbonate precipitation in the biomineralization of chromate and lead from an aqueous solution and soil. J Clean Produ. 2017;164:198–208. 10.1016/j.jclepro.2017.06.195

[82] Cuaxinque-Flores G, Aguirre-Noyola J, Hernández-Flores G, Martínez-Romero E, Romero-Ramírez Y, Talavera-Mendoza O. Bioimmobilization of toxic metals by precipitation of carbonates using *Sporosarcina luteola*: an in vitro study and application to sulfide-bearing tailings. Sci Total Environ. 2020;724:138124. 32268286 10.1016/j.scitotenv.2020.138124

[83] Achal V, Pan X. Characterization of urease and carbonic anhydrase producing bacteria and their role in calcite precipitation. Curr Microbial. 2011;62(3):894–902. 10.1007/s00284-010-9801-4 21046391

[84] Zain N, Kadir M. The Stabilisation of calcium carbonate vaterite phase via integration of mussel-inspired polydopamine. Inter Med Dev Technol Conf. 2017; 203–206.

[85] Hao Z, Bechtel H, Kneafsey T, Gilbert B, Nico P. Cross-scale molecular analysis of chemical heterogeneity in shale rocks. Sci Rep. 2018;7:1–9. 10.1038/s41598-018-20365-6 PMC5803189 29416052

[86] Hamedi A, Trotta F, Borhani Zarandi M, Zanetti M, Caldera F, Anceschi A, Nateghi M. In situ synthesis of MIL-100 (Fe) at the surface of Fe3O4 AC as highly efficient dye adsorbing nanocomposite. Intern J Mol Sci. 2019;20(22):5612. 10.3390/ijms20225612 PMC6888277 31717564

[87] Saracho A, Haigh K, Hata T, Soga K, Farsang S, Redfern A, Marek E. Characterisation of CaCO_3_ phases during strain-specific ureolytic precipitation. Sci Rep. 2020;23:1–2. 10.1038/s41598-020-66831-y PMC7311398 32576861

[88] Rodriguez-Navarro Cizer C, Kudłacz K, Ibañez-Velasco A, Ruiz-Agudo C, Elert K, Ruiz-Agudo E. The multiple roles of carbonic anhydrase in calcium carbonate mineralization. Cryst Eng Comm. 2019;21(48):7407–23. 10.1039/C9CE01544B

[89] Shafiu KA, Ismail M, Tengku T, Ibrahim A, Zakaria. Synthesis and characterisation of calcium carbonate aragonite nanocrystals from cockle Z shell powder (Anadara granosa). J Nanomat. 2013;2013:1687–4110.

[90] Hassan S, Fouda A, Radwan A, Salem S, Barghoth M, Awad M, Abdo A, El-Gamal M. Endophytic actinomycetes *Streptomyces sp*. mediated biosynthesis of copper oxide nanoparticles as a promising tool for biotechnological applications. J Biolog Inorg Chem. 2019;1(24):377–93. 10.1007/s00775-019-01654-5 30915551

[91] Lindskog S. Structure and mechanism of carbonic anhydrase. Pharmacol Ther. 1997;1(1):1–20. 10.1016/S0163-7258(96)00198-2 9336012

[92] Daskalakis M, Rigas F, Bakolas A, Magoulas A, Kotoulas G, Katsikis I, Mavridou A. Vaterite bio-precipitation induced by *Bacillus pumilus* isolated from a solutional cave in Paiania. Athens Greece. Intern Biodeter Biodeg. 2015;99:73–84. 10.1016/j.ibiod.2014.12.005

[93] Chada V, Hausner D, Strongin D, Rouff A, Reeder R. Divalent Cd and Pb uptake on calcite {10.14} cleavage faces An XPS and AFM study. J Colloid Interface Sci. 2005;288:350–60. 15927599 10.1016/j.jcis.2005.03.018

[94] Sdiri A, Higashi T. Simultaneous removal of heavy metals from aqueous solution by natural limestones. Appl Water Sci. 2013;3:29–39. 10.1007/s13201-012-0054-1

[95] Du Y, Lian F, Zhu L. Biosorption of divalent Pb, Cd and Zn on aragonite and calcite mollusk shells. Environ Pollut. 2011;159:1763. 21550150 10.1016/j.envpol.2011.04.017

[96] Zhang D, Ma Y, Feng H, Hao Y. Adsorption of Cr(VI) from Aqueous solution using carbon-microsilica composite adsorbent. J Chil Chem Soc. 2012;57:964–968. 10.4067/S0717-97072012000100002

[97] Wang L, Chen Y. Sequential precipitation of iron, copper, and zinc from wastewater for metal recovery. J Environ Eng. 2018. 10.1061/(ASCE)EE.1943-7870.0001480. 10.1061/(ASCE)EE.1943-7870.0001480

[98] Pang F, Teng S, Teng T, Omar A. Heavy metals removal by hydroxide precipitation and coagulation- flocculation methods from aqueous solutions. Water Qual Res J Can. 2009;44(2):174–82. 10.2166/wqrj.2009.019

